# Classical Data in Quantum Machine Learning Algorithms: Amplitude Encoding and the Relation Between Entropy and Linguistic Ambiguity

**DOI:** 10.3390/e27040433

**Published:** 2025-04-16

**Authors:** Jurek Eisinger, Ward Gauderis, Lin de Huybrecht, Geraint A. Wiggins

**Affiliations:** 1 Computational Creativity Lab, Vrije Universiteit Brussel, Pleinlaan 9, 1050 Elsene, Belgium; ward.gauderis@vub.be (W.G.); lin.de.huybrecht@vub.be (L.d.H.); 2 School of Electronic Engineering and Computer Science, Queen Mary University of London, London E1 4NS, UK

**Keywords:** quantum natural language processing, syntactic ambiguity, quantum machine learning

## Abstract

The *Categorical Compositional Distributional* (DisCoCat) model has been proven to be very successful in modelling sentence meaning as the interaction of word meanings. Words are modelled as quantum states, interacting guided by grammar. This model of language has been extended to density matrices to account for ambiguity in language. Density matrices describe probability distributions over quantum states, and in this work we relate the mixedness of density matrices to ambiguity in the sentences they represent. The von Neumann entropy and the fidelity are used as measures of this mixedness. Via the process of *amplitude encoding*, we introduce classical data into quantum machine learning algorithms. First, the findings suggest that in quantum natural language processing, amplitude-encoding data onto a quantum computer can be a useful tool to improve the performance of the quantum machine learning models used. Second, the effect that these encoded data have on the above-introduced relation between entropy and ambiguity is investigated. We conclude that amplitude-encoding classical data in quantum machine learning algorithms makes the relation between the entropy of a density matrix and ambiguity in the sentence modelled by this density matrix much more intuitively interpretable.

## 1. Introduction

**Figure 1:**
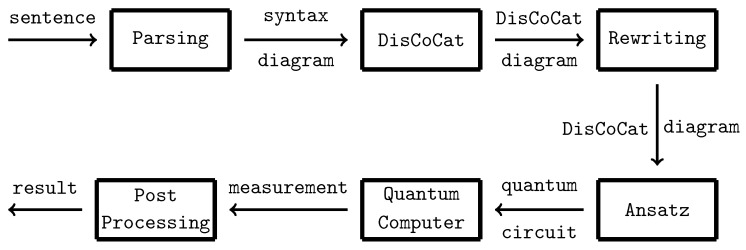
The training pipeline used in this work [3], showing the process to move from a sentence to a quantum circuit representing the meaning of this sentence. This quantum circuit is then executed on a quantum computer or simulations thereof. The training procedure amounts to adjusting the gate parameters in the quantum circuit based on the measurement results.

It is hypothesised that quantum computers will surpass classical computers in performance for certain tasks. In the context of quantum machine learning [1], quantum computers, often combined with classical models, are employed as machine learning frameworks. Quantum natural language processing (QNLP) leverages quantum machine learning models to represent meanings of words and sentences. Given that we are currently in the noisy intermediate-scale quantum (NISQ) era, quantum computers are not yet capable of outperforming classical computers on tasks involving large datasets. However, numerous approaches from the literature, which will be discussed in this work, demonstrate that concepts and principles derived from quantum theory are beneficial in natural language processing (NLP). These quantum-inspired methods offer more intuitive ways of reasoning about certain linguistic phenomena. A key focus of this study is the interpretability of machine learning algorithms, an area in which QNLP presents a distinct advantage over classical NLP. We investigate the relation between variations in the entropy of a quantum state, which represents a sentence in natural (human) language, and variations in the ambiguity of this sentence. To understand where this connection comes from, we first introduce the Categorical Compositional Distributional (DisCoCat) [2] model of language: words are modelled as quantum states, and the interaction of word meanings amounts to quantum states in different Hilbert spaces interacting with each other. These interactions can be captured by quantum circuits, which, when parameterised, are quantum machine learning models (*variational quantum circuits*) that are trained to predict sentence meaning. This training process involves adjusting the parameters of the parameterised gates, similar to neural networks. The training pipeline used in this work is depicted in Figure 1.

Ambiguity is modelled by omitting words from sentences, which results in probability distributions over different possible completions of partial sentences. Because quantum states represent sentences, we can measure the *von Neumann* entropy of these probability distributions. The entropy is related to the level of ambiguity in the modelled sentence.

The quantum machine learning model *learns* vector spaces in the training process. We investigate the interaction between these learned vector spaces and classically trained (word2vec) vector spaces, by applying *amplitude encoding*: a process to encode classical data onto quantum circuits, capturing the meanings of sentences. It is important to note that the use of amplitude encoding for mapping classical data onto a quantum computer in this context serves to examine the impact of classical data on the relationship between entropy and linguistic ambiguity. This study does not aim to analyse the broader effects of amplitude encoding in quantum machine learning models.

Secondly, we investigate the above connection between ambiguity and entropy with respect to the presence or absence of amplitude-encoded nouns in the learning process. The goal is to investigate to what extent the entropy of a density matrix representing sentence meaning measures the level of ambiguity in the represented sentence and how introducing classical data impacts this relation. We find that the cases in which amplitude encoding is employed display the hypothesised connection between ambiguity and entropy, while the non-amplitude-encoded approaches do not show this connection.

In Section 2, we give an overview of related work and address the background knowledge, including the DisCoCat model and edits connection to quantum computing and density matrices. We reproduce a study [3] in which quantum machine learning models are trained on a binary classification task in Section 3. Our research is grounded in this study, as we intend to modify the training procedure of the variational quantum circuits (VQCs) and utilise the trained models to explore the interpretability of the relationship between entropy and ambiguity in the subsequent sections. In Section 4, we apply amplitude encoding and compare the performance of the models to the models trained in the replication task (Section 3). In Section 5, the trained models are used to reason about ambiguity in sentences. The possibility of reasoning with trained models reflects the inherent *compositionality* of the quantum machine learning models [4]. By discarding qubits (modelling the process of leaving out words), we introduce ambiguity in quantum circuits representing sentences. We track the entropy for varying levels of ambiguity for both models in which amplitude encoding is used, and models in which it is not. Concretely, this paper makes three contributions:

1. Following Lorenz et al. [3], we train variational quantum circuits to learn meanings of words and sentences in a dataset. We explore the encoding of nouns on two qubits, in contrast to the one qubit in the original study, and we use an additional dataset, closely related to the one in the original study.
2. We investigate the effect of amplitude-encoding classical data on the models trained in the step before.
3. We then investigate the effect that classical data (introduced via amplitude encoding) has on the relation between the ambiguity in a sentence and the von Neumann entropy in the quantum state representing it.

## 2. Related Work

### 2.1. Quantum Natural Language Processing

Recent advancements in natural language processing (NLP) have predominantly centred on neural network-based approaches, with a strong emphasis on large language models (LLMs). These models leverage extensive datasets to capture linguistic structures and patterns, thereby achieving impressive results in various NLP tasks. However, their reliance on massive computational resources and the opaque nature of their decision-making processes present significant challenges in explainability and interpretability.

In contrast, the emerging field of quantum natural language processing (QNLP) introduces a fundamentally different perspective by using concepts from quantum theory to process linguistic information. Unlike classical models, which typically operate within a probabilistic framework, QNLP exploits the inherent superposition and entanglement properties of quantum systems to represent relationships in language. This approach holds promise for more efficient computations and potentially greater interpretability.

Initial research in QNLP has explored various quantum algorithms for text classification, sentiment analysis, and syntactic parsing, demonstrating promising results. Studies have highlighted how quantum circuits can efficiently encode linguistic structures, offering new pathways to address challenges faced by classical NLP models.

Despite its potential, QNLP remains in its nascent stages, with several open challenges. The limitations of current quantum hardware, including error rates and qubit coherence times, impose practical constraints on applications.

### 2.2. Theoretical Foundations of QNLP

The Categorical Distributional Compositional (DisCoCat) model of language, the basic mathematical framework our research is built on, was first proposed by Coecke et al. [2]. Lorenz et al. [3] connect this mathematical framework to the realm of quantum computing and present a mapping from DisCoCat diagrams to quantum circuits. These quantum circuits are machine learning models whose parameters are adjusted in the process of the model learning meanings of words and sentences.

The use of density matrices alongside the von Neumann entropy is well explored in QNLP. Density matrices are used to model ambiguity [5]. Meyer and Lewis [6] propose a framework called word2DM to learn density matrix embeddings. Leveraging this framework, Bruhn [7] combines word2DM with quantum computing. The learned density matrices are explicitly encoded on quantum circuits. Additionally, Hoffmann [8] learns the density matrix representations themselves from a language corpus. Pure state vectors representing words are learned individually. Thereafter, density matrices are explicitly constructed as probability distributions over the individual vectors and the entropy is investigated. Coecke [9] proposes the *DisCoCirc* framework, in which sentences are modelled by processes acting on density matrices representing words and sentences. These processes alter meanings of individual words and compositions thereof. Eisinger et al. [10] propose modelling syntactic ambiguity using probability distributions over completely positive maps that operate on density matrices representing sentence meaning.

To mathematically enable the DisCoCat framework to guide the composition of words represented by density matrices, Balkir et al. [11] introduce the compact closed category of *completely positive maps* (CPMs). The diagrams in which density matrices represent word meanings are drawn as *doubled* wires [12]. In these doubled diagrams, states can be *discarded*, amounting to “throwing the quantum state away” [13].

The current article follows work by Wijnholds [14], who formally models the semantic flow in sentences containing *verb phrase ellipsis* [15] and *parasitic gaps* [16] by using a *multimodal* extension of the *Lambek calculus* [17]. Wazni et al. [18] model verb phrase ellipsis by introducing *Fock spaces* [19], and copying mechanisms in language as *projections* from these Fock spaces.

We reproduce a binary classification task by Lorenz et al. [3], in which a machine learning model is trained to predict the category a sentence belongs to: either food (e.g., man prepares meal) or IT (e.g., woman debugs program).

### 2.3. The DisCoCat Framework

Language can be modeled through its distributional and compositional characteristics. The distributional hypothesis [20] suggests that words with similar meanings tend to appear in comparable contexts. Using the principle of compositionality, the meaning of sentences is derived from the meanings of their components, following grammatical rules. In the *Categorical Compositional Distributional* (DisCoCat) framework [2], the term *distributional* refers to assigning meaning to quantum states, while *compositional* pertains to the grammatical structure provided by the pregroup formalism. These two aspects are unified by *category theory*.

In the DisCoCat model, compositions of tensors are represented as *tensor networks* [21], which are realised as *quantum circuits* [22].

The *pregroup grammar* [23], a simplified version of the *Lambek calculus* [17], serves as the grammatical framework for composing quantum states that represent word meanings within the DisCoCat framework. Each word in a sentence is assigned an atomic type *p*, corresponding to its grammatical role. Two reduction rules guide the composition of quantum states:

(1) $$p^{l}\;\cdot \;p\to 1\;\;p\;\cdot \;p^{r}\to 1$$

If a sentence reduces to the canonical sentence type *s* upon multiplication of the types of its constituent words, it is considered grammatical:

(2) $$t_{\mathrm{sentence}}=\prod_{w}t_{w}\to s$$

By assigning types to each word in the sentence Alice plays guitar (Alice →n, plays$\to n^{r}\;\cdot \;s\;\cdot \;n^{l}$, and guitar→n) and applying the reduction rules, we obtain

(3) $$\mathrm{Alice plays guitar}:\;\;n\;\cdot \;\left(n^{r}\;\cdot \;s\;\cdot \;n^{l}\right)\;\cdot \;n\to 1\;\cdot \;s\;\cdot \;1\to s$$

This reduction shows that the sentence is grammatical.

The pregroup grammar is a *compact closed category* of pregroups, denoted Preg. As such, it serves as the formalism for guiding the composition of word meanings. Compact closed categories are linked to a diagrammatic language known as *string diagrams*: (4)
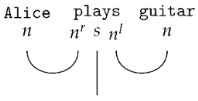

Words are modelled as tensors of different ranks within the compact closed category of vector spaces, known as FVect [24]. The DisCoCat framework arises as the Cartesian product between the two categories FVect and Preg.

DisCoCat’s morphisms are pairs of morphisms:

(5) (f:V→W,[p≤q])

where *f* is a linear map and [p≤q] is a pregroup partial order. DisCoCat’s tensor product is (V,p)⊗(W,q)=(V⊗W,p·q). The ranks of tensors representing words correspond to the grammatical types assigned to them.

In the above example, the words Alice and guitar are assigned vectors in the *noun space N*: ${\vec{v}}_{\mathrm{Alice}},{\vec{v}}_{\mathrm{guitar}}\in N$. The transitive verb plays, on the other hand, is a rank-three tensor: ${\vec{v}}_{\mathrm{plays}}\in N⊗S⊗N$, with the *sentence space S*.

**Figure 2:**
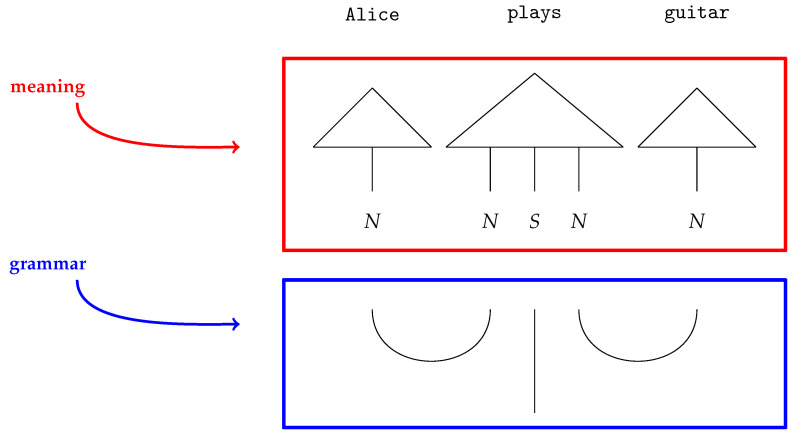
A DisCoCat diagram, composing tensors representing the meanings of words, guided by grammar.

String diagrams (*DisCoCat diagrams*) capture the composition of the tensors, with wires illustrating how tensors, represented by boxes, are composed (Figure 2). The DisCoCat model has been widely studied, with numerous extensions in various directions. It has been used in applications such as language translation [25], which led to the creation of *language circuits* [26]. Additionally, Coecke and Wang [27] explore the internal structure of words. Additionally, DisCoCat has found application in the domain of music [28], resulting in a quantum model for musical composition.

In Appendix A, a diagrammatic language for the Lambek calculus is presented, following Wijnholds [14]. This framework is utilised in Section 5 to construct DisCoCat diagrams.

A key concept in category theory, which facilitates the combination or deletion of information, is the *Frobenius algebra*. This structure, originally introduced by Frobenius [29] in group theory, appears within the category of finite-dimensional vector spaces FVect, and was later adopted in category theory [30].

A *Frobenius algebra* is defined as a tuple (X,Δ,ı,μ,ξ) within a symmetric monoidal category, where

(6) $$\begin{array}{cc} \Delta & :X\to X⊗X\;\;ı:X\to I\\ \mu & :X⊗X\to X\;\;\zeta :I\to X \end{array}$$

The two morphisms Δ and μ are represented by specific *spiders* in the ZX-calculus [31] and must satisfy the *Frobenius condition* [32]. Sadrzadeh et al. [32] utilise Frobenius deletion maps to model subject- and object-relative pronouns, such as in the sentences humans whom animals eat versus humans who eat animals. Wijnholds [14] suggests that the map Δ can be used to duplicate linguistic information. Consider the following sentence:

(7) Mary eats. She is hungry.

**Figure 3:**
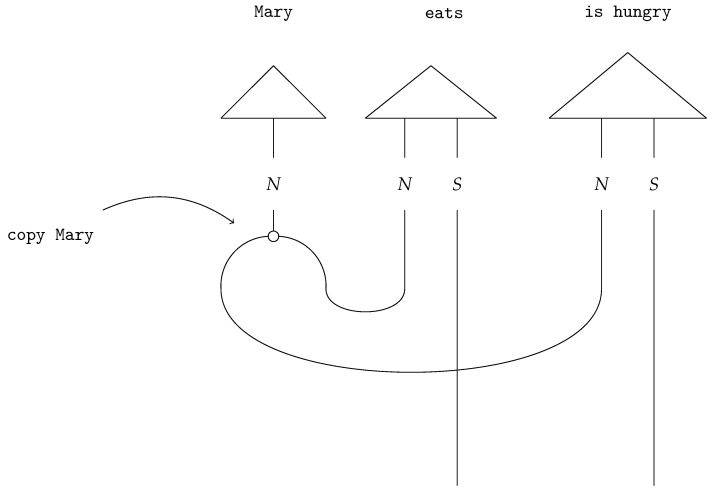
The meaning of the sentences Mary eats. Mary is hungry. as a pregroup diagram, after Wazni et al. [18], who use a different framework based on projections from Fock space.

Copying the word Mary and redirecting the information flow in the sentence (Figure 3) yields the correct interpretation (Mary eats. Mary is hungry.).

### 2.4. Quantum Computing

For a comprehensive introduction to quantum computing well beyond the brief overview given in this section, see, for instance, Nielsen and Chuang [33]. It is theorised that quantum computers should be able to outperform classical computers in certain tasks, such as Grover’s algorithm [34], a quantum search algorithm, and Shor’s algorithm [35] for prime factorisation. Currently, quantum computing is in the *noisy intermediate-scale quantum* (NISQ) era, characterised by the limited applicability of quantum computers due to high error rates and short qubit coherence times.

Quantum computing’s scope is not restricted to problems from physics. In fact, it has led to the development of a new field called *quantum machine learning* (QML), which intersects with quantum natural language processing (QNLP).

Quantum computing involves performing computations on *qubits*, which are the quantum equivalent of classical *bits*. The computation process consists of a sequence of manipulations of these qubits. While a classical bit can only take values 0 or 1, a qubit can naturally assume values in between as well. The quantum state |q〉 of a qubit is

(8) |q〉=α|0〉+β|1〉

where α and β are constants in C, and ${\left|\alpha \right|}^{2}+{\left|\beta \right|}^{2}=1$.

**Figure 4:**
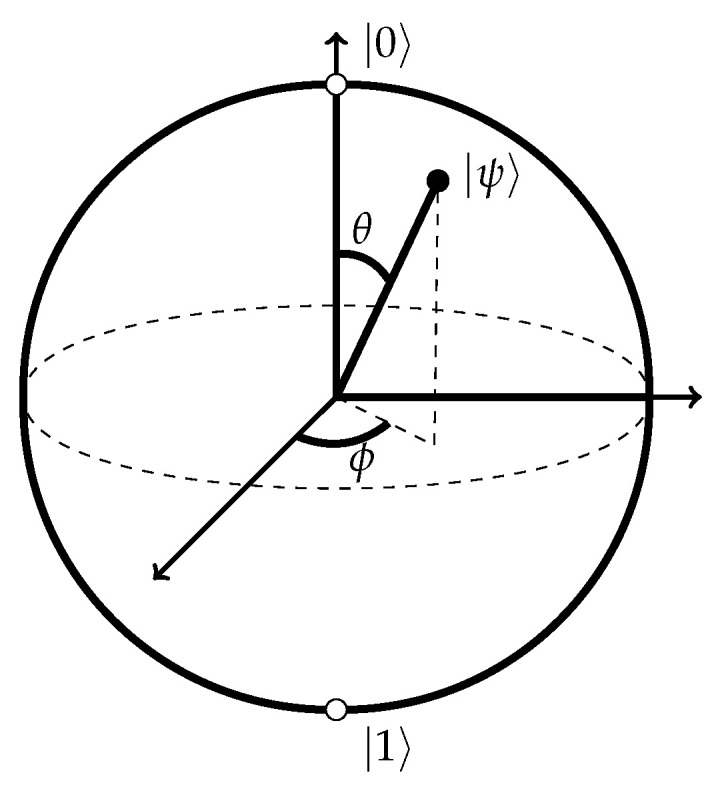
The Bloch sphere visualisation of qubit state vectors, where the black dot represents the state vector |ψ〉 of a qubit on the Bloch sphere.

Quantum gates serve as the fundamental building blocks of quantum circuits and are represented by unitary matrices *U*:

(9) $$U^{†}U=I=UU^{†}$$

where † denotes the Hermitian adjoint. The state vector of a qubit can be visualised on the *Bloch sphere*. Single-qubit quantum gates rotate the qubit state vectors on the sphere (Figure 4). The most common quantum gates rotate the qubit state around the *x*-, *y*-, or *z*-axis of the Bloch sphere by an angle of π. These gates, called Pauli-*X*, -*Y*, and -*Z* gates, respectively, are represented by the Pauli matrices

(10) $$X\;=\;\left(\begin{array}{cc} 0 & 1\\ 1 & 0 \end{array}\right)\;\;Y\;=\;\left(\begin{array}{cc} 0 & -i\\ i & 0 \end{array}\right)\;\;Z\;=\;\left(\begin{array}{cc} 1 & 0\\ 0 & -1 \end{array}\right)$$

In quantum circuit notation, qubits correspond to vertical lines, and gates are applied to the qubits from left to right in the circuit. The Pauli gates are(11)
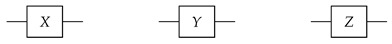

The matrix representations of generalised one-qubit rotation gates are

$$R_{X}\left(\theta \right)\;=\;\left(\begin{array}{cc} \cos(\theta /2) & -i\sin(\theta /2)\\ -i\sin(\theta /2) & \cos(\theta /2) \end{array}\right)\;R_{Y}\left(\theta \right)\;=\;\left(\begin{array}{cc} \cos(\theta /2) & -\sin(\theta /2)\\ \sin(\theta /2) & \cos(\theta /2) \end{array}\right)\;R_{Z}\left(\theta \right)\;=\;\left(\begin{array}{cc} e^{-i\theta /2} & 0\\ 0 & e^{i\theta /2} \end{array}\right)$$

The Hadamard gate H, characterised by(12)
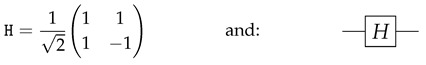

operates on the two basis states |0〉 and |1〉 as follows:

$$H|0\rangle =\frac{1}{\sqrt{2}}\left(|0\rangle +|1\rangle \right):=|+\rangle \;H|1\rangle =\frac{1}{\sqrt{2}}\left(|0\rangle -|1\rangle \right):=|-\rangle$$

It performs a basis change by flipping the x-axis and z-axis.

A key example of a two-qubit gate is the *controlled NOT* (CNOT) gate:

(13) $$\mathrm{CNOT}\;=\;\left(\begin{array}{cccc} 1 & 0 & 0 & 0\\ 0 & 1 & 0 & 0\\ 0 & 0 & 0 & 1\\ 0 & 0 & 1 & 0 \end{array}\right)$$

The gate is called the *controlled* NOT because it applies a NOT operation to the second qubit only if the first qubit is in state |1〉. In the circuit notation(14)
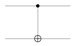

the black dot indicates the *control* qubit, while the white dot with a cross represents the NOT gate.

Usually, the $N_{q}$ qubits in a quantum circuit are initialised to the zero-state:

(15) $$|\psi \rangle =\underset{N_{q}\;\mathrm{times}}{\underset{︸}{|0\rangle ⊗|0\rangle ⊗\ldots ⊗|0\rangle }}=|00\ldots 0\rangle$$

The manipulation of qubits is achieved by sequentially applying the gates in the quantum circuit. The machine learning models used in Section 3 are quantum circuits that are trained to predict meanings of sentences. In the training process, parameters of parameterised gates are adjusted based on a loss function.

In quantum circuits, qubits are typically *entangled*. A state is considered entangled when it consists of multiple states that cannot be described independently. When an entangled qubit is examined in isolation, its state is known as a *mixed* state. Observing one qubit in an entangled system causes the states of the remaining qubits to collapse.

In the context of quantum natural language processing (QNLP), the concept of entanglement plays a key role in modelling the precise relationship between quantum states that represent the meanings of different words.

### 2.5. From Linguistics to Quantum Circuits

**Figure 5:**
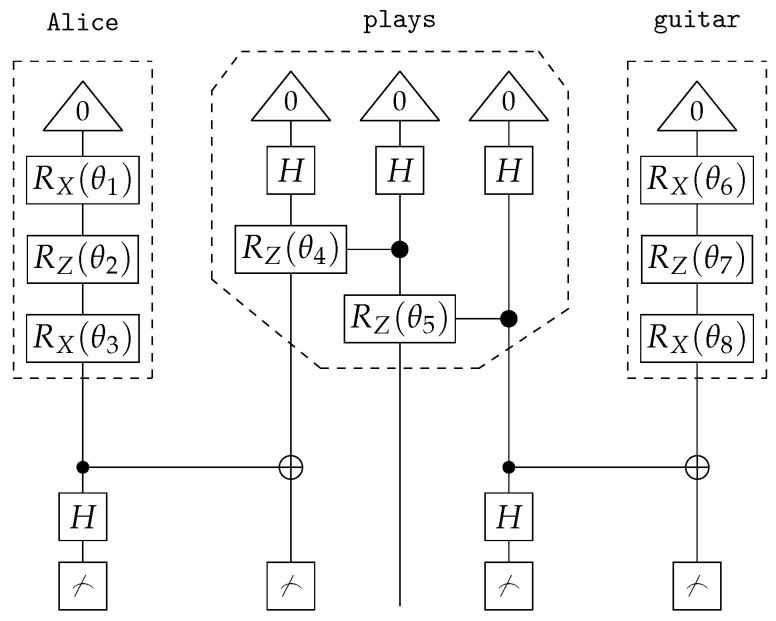
Example circuit encoding the meaning of the sentence Alice plays guitar. Qubits are represented by vertical lines rather than horizontal lines in the usual quantum circuit notation, to emphasise the connection to DisCoCat diagrams. The combination of Hadamard-, CNOT-, and measurement gates correspond to the cup-shaped wires in DisCoCat diagrams. The translation of DisCoCat diagrams to quantum circuits is performed in such a way that the measurement has to yield 0 for the circuit to capture the sentence correctly.

If the DisCoCat framework and quantum computing are combined, variational quantum circuits (VQCs) arise, that capture the meaning of words or sentences (Figure 5 displays such a quantum circuit for the example sentence of Alice plays guitar).

Each word in the sentence is associated with its own sub-circuit, and in Figure 5, one qubit encodes the meaning of both the noun and sentence spaces. For the circuit to correctly represent the meaning of the sentence, the measurement gates (Figure 5) must yield the measurement |0〉. In some cases, this measurement is depicted as a bra-state: (16)
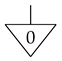

This notation indicates that the measurement should be interpreted as a *test* to determine if the qubit is in the |0〉 state, which is ensured through a process called *post-processing*. The transition from DisCoCat to quantum circuits is formalised by Lorenz et al. [3] and Coecke et al. [5], who describe it in terms of *tensor networks* [21] and the field of *quantum picturialism* [36]. These fields originated from a diagrammatic notation introduced by Penrose et al. [37], which offers a way to reason about tensors in quantum mechanics. Additionally, the choice of *ansatz* plays a critical role. It determines how individual qubits represent the meaning of words and how qubits representing word meaning interact with each other. For example, a noun’s meaning can be captured by one or two qubits, making the complex noun space two- or four-dimensional, respectively.

Following Lorenz et al. [3], we adopt the IQP-ansatz [38] and encode both the sentence and noun meanings onto a single qubit (Figure 5). We employ a binary classification task, where words in sentences are the input, and the output consists of quantum states representing sentence meanings corresponding to categories (represented by |1〉,|0〉).

In this study, classical data are encoded onto a quantum circuit using *amplitude encoding* (for an overview of different encoding approaches, see https://pennylane.ai/qml/glossary/quantum_embedding/ (accessed on 27 July 2024)). Amplitude encoding, as the name suggests, involves encoding classical data onto the amplitudes of quantum states. This technique is commonly used to give a variational quantum algorithm (VQA) an initial advantage, as noted by, for example, Truger et al. [39].

This approach is particularly useful due to the existence of *barren plateaus* [40], where the gradients in the optimisation landscape of a variational quantum eigensolver (VQE) vanish exponentially with the number of qubits in the model. By pretraining a VQC [41], the complexity of training on quantum hardware can be minimised.

Generally, for an *n*-qubit quantum state, we have

$$|\psi \rangle =\sum_{i=1}^{N}x_{i}|i\rangle$$

we can now encode an $N=2^{n}$-dimensional vector onto this quantum state by replacing the factors $x_{i}$ with the *i*-th element of a classical vector. For a two-qubit system and some four-dimensional vector $x={\left(\begin{array}{c} x_{1},x_{2},x_{3},x_{4} \end{array}\right)}^{T}$, we can then associate this vector with the quantum state

$$\psi_{x}=x_{1}|00\rangle +x_{2}|01\rangle +x_{3}|10\rangle +x_{4}|11\rangle$$

and successfully encode the components of the vector onto a quantum state. The last step in this procedure entails normalising this vector by dividing it by its magnitude.

Note that amplitude-encoding data onto quantum circuits is different from the approach of using quantum states representing categories that the model is trained to predict. Quantum states representing classical categories are *learned* by the model rather than classical data being explicitly *encoded* onto the quantum circuit.

We employ the *binary cross-entropy* loss function (Lorenz et al. [3] explain the optimisation procedure of the parameters). For quantum circuits, the output is probabilistic and the probability distribution must be reconstructed through multiple measurements. Thus, obtaining the gradient for optimisation processes involving quantum circuits is a non-trivial task. For this reason, the *Simultaneous Perturbation Stochastic Approximation* algorithm [SPSA] [42] is used, which is the most common method for approximating gradients for variational quantum circuits [3,43,44].

### 2.6. Density Matrices

Density matrices play a crucial role in the context of QNLP because they can model ambiguity in language and capture the hierarchical relationships between word meanings [45]. We focus on the former aspect.

A density matrix ρ is a probability distribution over quantum states:

(17) $$\rho =\sum_{i}p_{i}|\psi_{i}\rangle \langle \psi_{i}|$$

where $|\psi_{1}\rangle ,\;|\psi_{2}\rangle ,\;\ldots$ are pure quantum states, and $p_{1},\;p_{2},\;\ldots$ are the corresponding probabilities. It is a positive semi-definite, Hermitian operator with trace one. Density matrices are referred to as *mixed states*, whereas state vectors represent *pure states*.

The density matrix is used to describe the state of systems entangled with other quantum states, or when information about the initialisation of systems is missing.

Consider an operator *A* in a system described by the density matrix ρ. The expected value of *A* is

(18) $$\langle A\rangle =\mathrm{Tr}\left(\rho \;A\right)$$

The expectation value for a pure state (|ψ〉) case is

(19) 〈A〉=〈ψ|A|ψ〉

The Schrödinger–HJW theorem [46] (The Schrödinger–HJW theorem is a special case of the *Stinespring dilation* [36]) states that any mixed state ρ can be *purified*, by representing it as the *partial trace* of a *pure state* $\psi_{12}$ in a composite Hilbert space $H=H_{1}⊗H_{2}$:

(20) $$\rho ={\mathrm{Tr}}_{2}\left(|\psi_{12}\rangle \langle \psi_{12}|\right)$$

where ${\mathrm{Tr}}_{2}$ is the *partial trace* over $H_{2}$. Here, ρ is a *reduced density matrix*.

**Figure 6:**
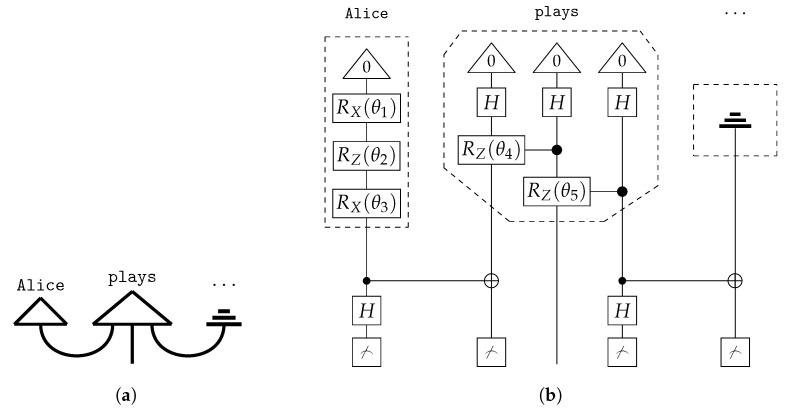
The diagram (**a**) and the quantum circuit (**b**) encoding the meaning of the sentence Alice plays …, where the three dots indicate that the respective word is missing from the sentence. The translation from the diagram to the quantum circuit is explained in the main text. Quantum gates in the dashed boxes represent meanings of words (the states Alice and plays in diagram (**a**)), while the quantum gates below represent the cup-shaped wires in diagram (**a**).

In the current article, we use the *discarding* effect to explicitly construct mixed states from pure states. *Discarding* a qubit amounts to tracing out its corresponding Hilbert space [36]. The discarding map(21)
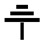

can be used in a composite diagram (Figure 6a), which itself corresponds to a quantum circuit (Figure 6b).

The *von Neumann entropy* measures the mixedness of a quantum state ρ. It serves as the quantum theoretical counterpart to the *Shannon entropy* [47], which measures *uncertainty* or average *information content*. The von Neumann entropy [48] is

(22) $$S_{\mathrm{Von}\mathrm{Neumann}}\;=-\mathrm{Tr}\left(\;\rho \ln\rho \;\right)$$

where ρ is a density matrix. The von Neumann entropy is bounded between 0, for pure states, and ln(d), for completely mixed states, where *d* is the dimension of the Hilbert space.

In this study, the von Neumann entropy is employed to analyse the information and uncertainty encoded within quantum circuits that represent words and sentences. Furthermore, the *fidelity* is utilised to quantify the similarity between two density matrices. The fidelity between two density matrices ρ and σ is defined as

(23) $$F\left(\rho ,\sigma \right)=\mathrm{Tr}{\left(\sqrt{\sqrt{\rho }\sigma \sqrt{\rho }}\right)}^{2}$$

Balkir et al. [11] argue that the fidelity is suitable as a measure for the comparison of density matrices.

In the study of language, density matrices serve as a tool for modelling both ambiguity and hierarchical relationships among words [5]. For instance, the word bank can denote a financial institute, a river bank, or a computer memory bank. The existence of multiple interpretations for a single word characterises it as *ambiguous*. To capture this ambiguity, a density matrix can be utilised to represent a probability distribution over the pure states corresponding to the various meanings of the word:

(24) $$\begin{array}{cc} \rho_{\mathrm{bank}} & =\alpha \;|{\mathrm{bank}}_{\mathrm{river}}\rangle \langle {\mathrm{bank}}_{\mathrm{river}}|\\ \; & +\beta \;|{\mathrm{bank}}_{\mathrm{finance}}\rangle \langle {\mathrm{bank}}_{\mathrm{finance}}|\\ \; & +\gamma \;|{\mathrm{bank}}_{\mathrm{memory}}\rangle \langle {\mathrm{bank}}_{\mathrm{memory}}| \end{array}$$

where α,β, and γ are positive real numbers summing to one.

Density matrices corresponding to other words within a given context interact with the previously defined density matrices, thereby refining the overall meaning of the sentence. For example, when a contextually related word such as fish appears alongside riverbank, it aids in disambiguating the meaning of $\rho_{\mathrm{bank}}$. A density matrix comprises a series of weighted projection operators. Through the composition of the density matrices $\rho_{\mathrm{fish}}$ and $\rho_{\mathrm{bank}}$, the fish meaning selects the riverbank interpretation from $\rho_{\mathrm{bank}}$, under the assumption that the state vectors corresponding to the various meanings—riverbank, financial bank, computer memory bank—are mutually orthogonal.

The pure eigenstates of the density matrix are typically constructed from more fundamental words. To capture these meanings, high-dimensional *count-based* vector spaces are frequently employed, with typical models trained using approximately 1000 basis words. These vector spaces are generated based on *context windows* surrounding the target word [49].

The transition from state vectors to density matrices in the representation of word meanings is formally described by Piedeleu et al. [12] as *doubling*. In diagrammatic representations, this transformation is visually depicted by using *thicker* wires to signify the shift to density matrices.

Density matrices serve as a means to model probability distributions over both *word meanings* and *sentence meanings*.

An essential concept in this context is measurement. As the density matrix evolves through the application of quantum gates, it is further altered by the measurement process. This process is mathematically represented by a measurement projector acting on the density matrix. The operation of a measurement projector $P_{x_{A}}$ is given by

(25) $$P_{x_{A}}=|x_{A}\rangle \langle x_{A}|⊗1_{B}$$

In a composite system $H_{AB}$, for $\left\{|x_{A}\rangle \right\}$ with a measurement basis of *A*, the effect of measuring is

(26) $$\rho^{\prime }=\frac{\mathrm{Tr}\;\left(P_{x_{A}}\rho P_{x_{A}}\right)}{\mathrm{Tr}(\rho P_{x_{A}})}=\frac{\langle \;x_{A}\;\left|\;\rho \;\right|\;x_{A}\;\rangle }{\mathrm{Tr}(\rho P_{x_{A}})}$$

A new normalised density matrix ($\mathrm{Tr}(\rho^{\prime })=1$ ) is produced.

## 3. The Underlying Replication Task

Lorenz et al. [3] investigated three fundamentally different grammatical models of combining word meaning and evaluated the performance of these models in two different binary classification tasks. Their task was to categorise sentences into the semantic categories food or IT using the dataset introduced in Section 3.1.

Lorenz et al. [3] use the Tket compiler [50], integrated into Lambeq [51] to simulate quantum hardware on a classical computer. The Tket model closely resembles a quantum computer and uses pytket (https://pypi.org/project/pytket/ (accessed on 12 August 2024)) to perform noisy, architecture-aware, *shot-based* simulations of a quantum computer, which can be run on real quantum hardware. The term *shot-based* refers to running the model numerous times to obtain an estimate of the probability distribution. We also use this model, alongside two further options:

1. NumPy model:
  Uses the Python library NumPy (https://numpy.org (accessed on 14 August 2024)). Quantum circuits are converted to tensor networks. The SPSA optimiser is used to estimate the gradient. The simulation is noiseless. The model cannot be run on real quantum hardware.
2. pennylane model:
  Uses the python libraries pennylane (https://pennylane.ai/ (accessed on 12 August 2024)) and PyTorch (https://pytorch.org/ (accessed on 12 August 2024)). Both state vector simulations and density matrix simulations can be performed. The pennylane model uses exact backpropagation, in contrast to the NumPy model that uses the *Simultaneous Perturbation Stochastic Approximation* (SPSA) function. This model can be run on real quantum hardware.

The Tket model is the main model in our work, while the pennylane and NumPy models serve as baselines.

Meanings of nouns are encoded on both one and two qubits in separate approaches, whereas Lorenz et al. [3] encode the meaning of nouns on one qubit only. We adopt the best-performing model according to Lorenz et al. [3], which is the model in which three layers of the IQP-ansatz are chosen.

### 3.1. Datasets

Two datasets (the datasets and code are available at https://github.com/jurekjurek/Classical-Data-in-Quantum-Machine-Learning (accessed on 15 February 2025)) are used in the current work. Example sentences in the first dataset [3], belonging to the categories food and IT, respectively, are

(27) $$\begin{array}{cc} \; & \mathrm{Skillful}\;\mathrm{man}\;\mathrm{prepares}\;\mathrm{sauce}\\ \; & \mathrm{Woman}\;\mathrm{runs}\;\mathrm{application} \end{array}$$

The vocabulary size of this dataset is 17 and the total number of sentences is 130, with 65 sentences per category. The dataset (Appendix C) has four ambiguous words (shared between the sentences of the two categories).

The second dataset is an extension of the first one: non-ambiguous subjects are used, which we briefly explain the motivation for in the following. In Section 5, ambiguity is introduced in sentences by forgetting words, as in the following example:

Person prepares …

where the three dots indicate a missing word. By introducing non-ambiguous subjects to the dataset, the sentence can be disambiguated [14]:

Person prepares …  and chef does too.

Chef is a non-ambiguous word, disambiguating the sentence.

To obtain the second from the first dataset, the word woman is replaced by the word chef if it is used in a sentence belonging to the category food. If it is used in a sentence belonging to the category IT, woman is replaced by programmer, and the same procedure is applied to replace the word man. The size of the vocabulary (17) and the number of sentences (65 per category) are the same as in the original dataset above. However, the number of ambiguous words that are shared between the categories is now only two (person and prepares), rather than four in the original dataset. We will refer to the first dataset as the *original dataset* and to the second dataset as the *new dataset*.

When training a machine learning model, the dataset is divided into three subsets: *test*, *train*, and *validation* sets [52]. The train set is used to optimise the model’s parameters, while the validation set is employed during training primarily to mitigate overfitting. The model does not adjust its parameters based on the validation set; instead, this set helps assess the model’s ability to generalise to unseen data. The test set is reserved for the final evaluation of the model’s performance. Thus, when reporting the final accuracy, it reflects the model’s performance on the unseen test set. In our case, we allocate 80% of the data to the train set and 10% each to the test and validation sets.

### 3.2. The Experiment

The models are trained on both datasets, and for each model the encoding of the noun meaning on both one and two qubits is investigated (referred to as the *one-qubit approach* and *two-qubit approach*, respectively). The evolution of the training and validation loss through the epochs is reported. The accuracy, Cohen’s kappa [53], as well as the $F_{1}$-score [54] are considered as measures of the model’s performance. The approach of encoding noun data onto two qubits and training models on the new dataset are an extension of the experiment by Lorenz et al. [3]. Note that neither the performance of the model in terms of the employed metrics, nor the convergence behaviour of the model, is directly correlated with the interpretability of the results, as investigated in Section 5. During training, the model converges to certain representations of word meaning. However, the quality of these representations, in terms of their interpretability concerning the connection between entropy and ambiguity (Section 5), is not explicitly captured by the performance metrics. Nonetheless, we expect a relation between the training performance and the interpretability: the better the performance, the more closely the model learns the training data. While we implicitly guide the model toward learning interpretable representations, we cannot explicitly enforce this during training.

Therefore, in Section 5, we investigate the effect that classical data have on the relation between entropy and linguistic ambiguity for *individual models*, rather than averaging the entropy values over sets of models. In this and the subsequent section, we present the loss curves and performance metrics for the models whose parameters are utilised in Section 5. It is important to note that our analysis does not aim to investigate the general training behaviour of these models, but rather relates training behaviour and interpretability. A more general investigation of the training behaviour of these models, particularly involving classical data (via amplitude encoding, Section 4), would entail averaging performance metrics and loss curves over multiple runs, each with a random parameter initialisation. The randomness in the initial parameters might lead to substantial variation in the individual loss curves and performance metrics due to different convergence of the models. The linear (Section 3.2.1) optimisation process of VQCs (in contrast to the non-linear optimisation of neural networks) might lead to, e.g., varying sensitivity to initial parameters. At present, we are not aware of a method to determine, or even estimate, the number of runs required for averaging (i.e., a threshold at which the variation among different loss curves is appropriately captured in the mean value). While the resulting loss curves may provide a more general representation of the model’s optimisation behaviour, they may also hide meaningful distinctions when comparing different encoding approaches (e.g., encoding noun meaning using one versus two qubits, or employing amplitude encoding). As this comparison constitutes the primary focus of our research, we prioritise the analysis of individual models.

#### 3.2.1. Tket Model

**Figure 7:**
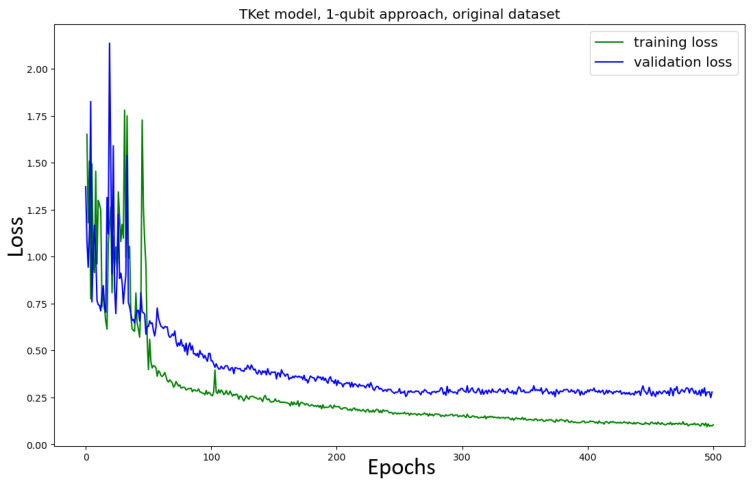
Loss curves for training and validation data for the Tket model with noun meanings encoded on one qubit. Metrics: accuracy = 0.900; κ= 0.867, $F_{1}$-score = 0.933.

First, the Tket model is trained on the *original* dataset, where the one-qubit approach is used (Figure 7). Although there is noise, the model converges after around 250 epochs, following the findings of Lorenz et al. [3]. After these 250 epochs, the training loss continues to decrease very slowly, while the validation loss stays constant. This behaviour is addressed in the discussion below.

**Figure 8:**
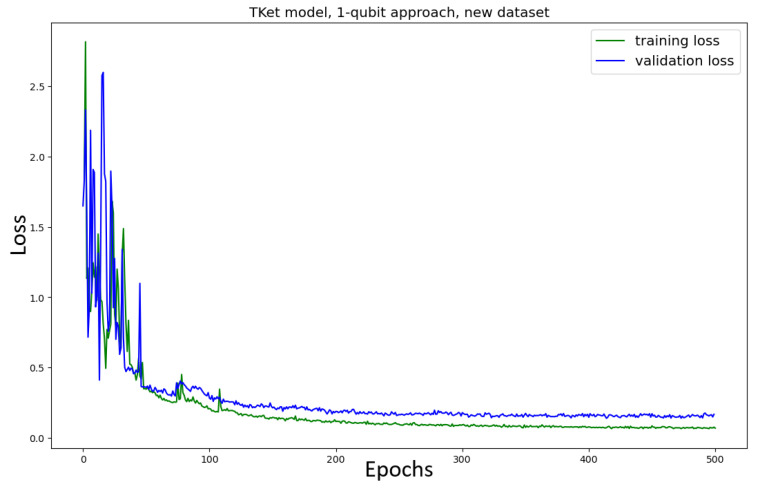
Loss curves for the Tket model with noun meaning encoded onto one qubit for new dataset. Metrics: accuracy = 0.967; κ = 0.867; $F_{1}$-score = 0.938.

Training the Tket model on the *new* dataset using the one-qubit approach (Figure 8) results in smooth convergence, similar to the model trained on the original dataset. A slightly (≈7%) higher accuracy can be reported for the model trained on the new dataset and a slightly faster convergence (200 epochs rather than 250). Here, we also see the effect of the model’s training loss slightly decreasing while the validation loss stays constant (after around 200 epochs).

**Figure 9:**
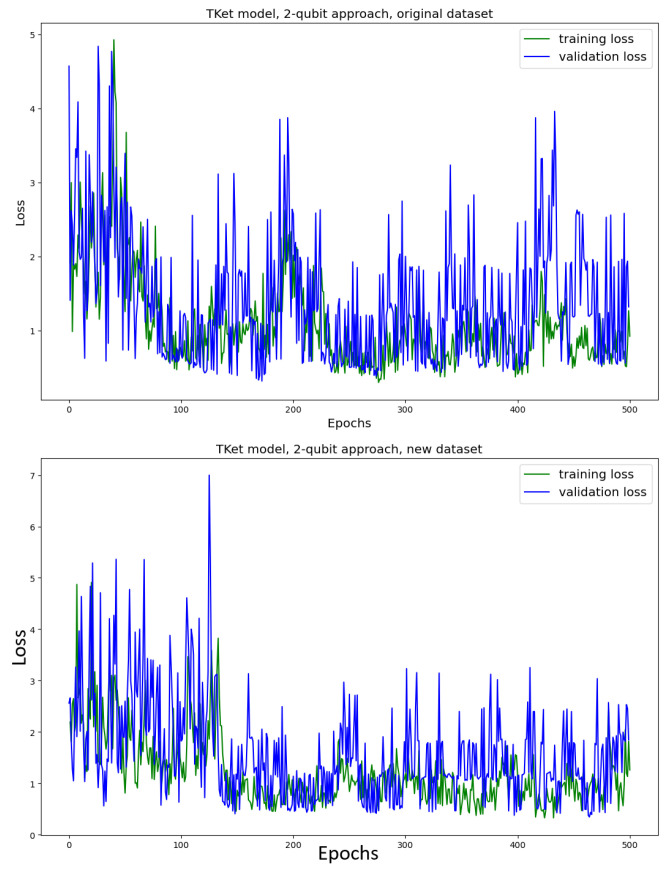
Loss curves for training and validation data for the Tket model with noun meanings encoded on two qubits using the original dataset (**top**) and the new dataset (**bottom**). Metrics, top: accuracy = 0.783, κ = 0.800, $F_{1}$-score = 0.903. Metrics, bottom: accuracy = 0.816, κ = 0.833, $F_{1}$-score = 0.759.

The Tket model is then trained in the *two-qubit* approach on the original and new datasets (Figure 9). The model trained on the *original* dataset shows no convergence, together with a significant drop in accuracy (≈13% decrease in accuracy) as compared to the one-qubit approach (Figure 7). Similar behaviour can be seen for the Tket model trained on the *new* dataset, where the convergence is slightly better (Figure 9, bottom) compared to the original dataset (Figure 9, top). However, the drop in accuracy compared to the one-qubit approach is substantial (≈16%).

Discussion

The effect of the Tket model performing worse when the noun meaning is encoded onto two qubits is likely due to the model’s search space increasing. Qubits are added to the corresponding quantum circuits, which results in the Hilbert space spanned by these qubits having a higher number of dimensions. The rising complexity in the search space results in the model’s inability to find a solution and converge. Because the Tket model trained on the new dataset performs better for both the one- and two-qubit approaches, we argue that the model trained on the new dataset can navigate the corresponding search space more effectively. This might be due to there being fewer ambiguous words in the new dataset. By providing the word, e.g., chef, the model has to learn the mapping from this word only to the category food, while for the words man and woman, the model learns a mapping to both categories food and IT for each of the words. This means that the corresponding sub-circuits encoding the meaning of the verbs and adjectives have to be able to generalise better.

The training loss decreases while the validation loss is constant in the one qubit approach, both for the original and the new datasets (Figure 7 and Figure 8). This is not a problem. The validation loss being constant does not imply that the model loses its ability to generalise, neither does it imply that the model’s performance improves. If the validation loss were to increase, this effect would be called *overfitting*, which in the classical sense describes the phenomenon of the model learning the training set too specifically while losing its ability to perform well on unseen data. In this case, one often sees the training loss decrease, while the validation loss (an indicator of how well the model performs on unseen data) increases. This often happens if the model is learning noise in the training dataset. Furthermore, overfitting is in many cases due to the model being too complex for the task at hand, which means that the number of parameters in the model allows for much more complex connections to be learned than the task requires.

The parameter search space in the case of a variational quantum circuit is *linear*. Individual parameterised sub-circuits are learned; these are combined, according to grammar formalisms, in a linear manner. The model’s prediction is a linear combination of learned quantum states. In contrast, the search space of a neural network (of sufficiently high complexity) is *non-linear*. This ability of the neural network to learn highly non-linear relations is one of the main reasons that the effect of overfitting is a prominent problem when using neural networks, subsequently leading to the training data being mapped too specifically. Mitarai et al. [55] argue that, in contrast to neural networks, the unitary nature of the transformations in the process of learning VQCs leads to the mitigation of overfitting. While the effect of overfitting still exists due to noise in the data and the limited sizes of datasets, the linear nature of the VQC does not allow for non-linearly fitted noise in the training data. Thus, one has to be careful in translating the concept of overfitting between classical machine learning and quantum machine learning and keep in mind the contrast between the linearity of Hilbert spaces and the non-linearity of search spaces learned by deep neural networks.

#### 3.2.2. NumPy and Pennylane Models

**Figure A3:**
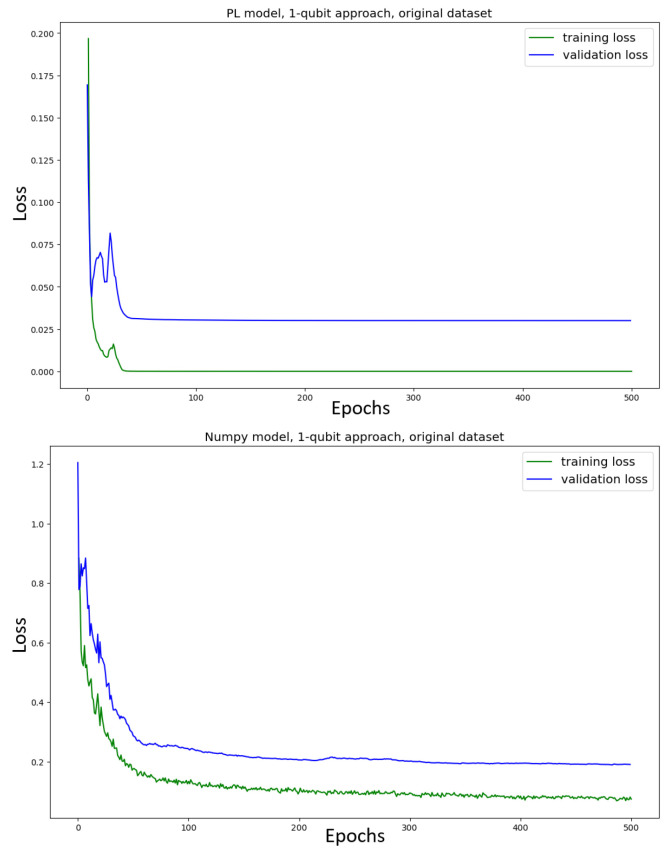
Loss curves for training and validation data for the pennylane (**left**) and NumPy (**right**) models, with noun meanings encoded on one qubit using the original dataset. Metrics, left: accuracy = 0.967, κ = 1.00, $F_{1}$-score: 1.00. Metrics, right: accuracy = 0.900, κ = 0.867, F_1_-score = 0.938.

**Figure A5:**
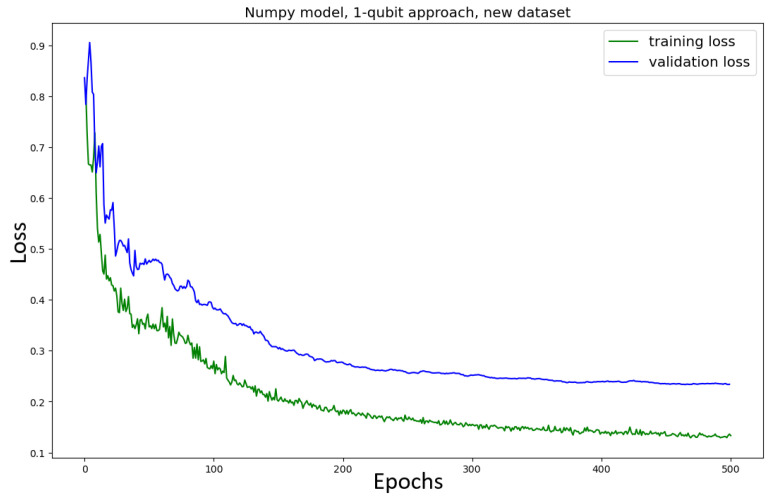
NumPy model, trained over 500 epochs, with noun meaning encoded onto one qubit on the new dataset. Metrics: accuracy = 0.90; κ = 0.87; F_1_-score = 0.94.

**Figure A6:**
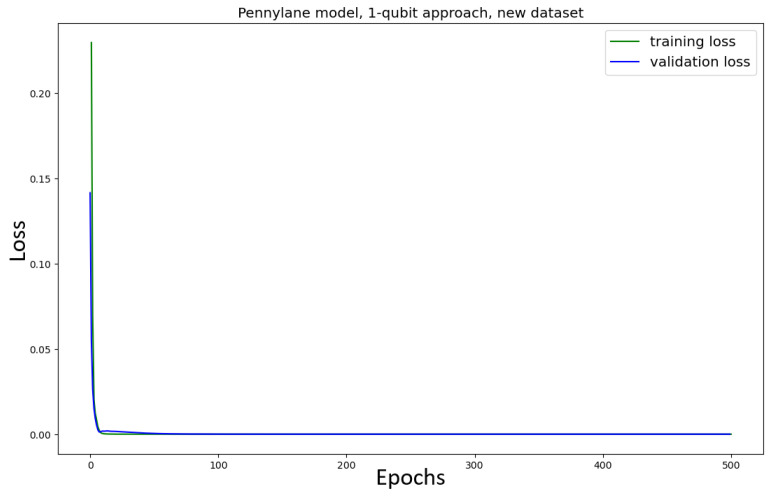
pennylane model, trained over 500 epochs, with noun meaning encoded onto one qubit on the new dataset. Metrics: accuracy = 0.97; κ = 1.0; F_1_-score = 1.0.

**Figure A7:**
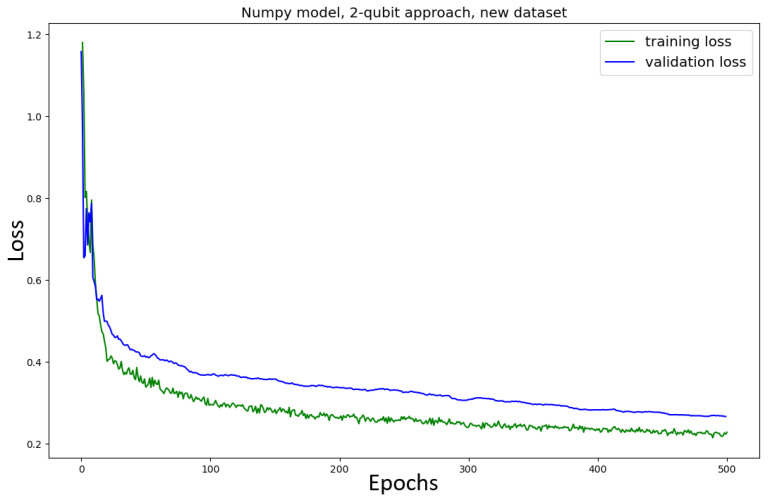
NumPy model, trained over 500 epochs, with noun meaning encoded onto two qubits on the new dataset. Metrics: accuracy = 0.80; κ = 0.60; F_1_-score = 0.79.

**Figure A8:**
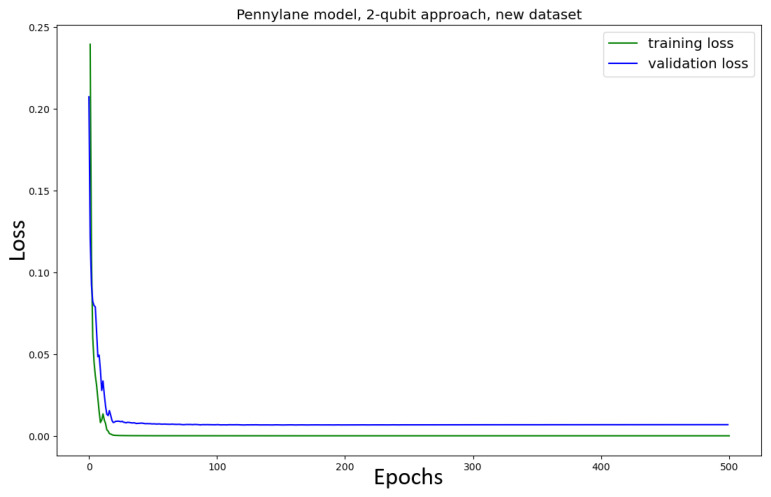
pennylane model, trained over 500 epochs, with noun meaning encoded onto two qubits on the new dataset. Metrics: accuracy = 1.0; κ = 1.0; F_1_-score = 1.0.

We note that both models converge smoothly, so we restrict ourselves to collecting the performance metrics for the different datasets, as well as the one- and two-qubit approaches, in Table 1. The pennylane and NumPy models are trained on the original dataset (Figure A3). The loss curves of the pennylane and NumPy models for the new dataset are depicted in Figure A6 (pennylane, one-qubit approach), Figure A8 (pennylane, two-qubit approach), Figure A5 (NumPy, one-qubit approach), and Figure A7 (NumPy, two-qubit approach). We restrict the discussion to the original dataset. For the one-qubit approach, while the pennylane model performs significantly better (≈7% more accurate, $F_{1}$- and κ-scores of 1) than the Tket model (Figure 7), the NumPy model performs very similarly to the Tket model.

For the two-qubit approach, as indicated by the metrics, the pennylane model’s predictions are perfect (accuracy, κ, as well as $F_{1}$-score are 1.0), considerably outperforming the Tket model. The performance of the NumPy model is very similar to that of the Tket model (Figure 9).

Discussion

The reason the pennylane model performs so well is twofold. Firstly, the pennylane model, and also the NumPy model, is trained without noise, while the Tket model is trained with noise. Secondly, the model is trained using exact backpropagation (using PyTorch) to determine the gradient in the optimisation procedure, while the NumPy model uses the SPSA algorithm, which estimates the gradient. This results in the pennylane model’s updating mechanism being more precise, ultimately leading to faster convergence and better predictions.

The similarity in performance between the NumPy and the Tket models is likely due to the similar underlying updating mechanisms.

## 4. Amplitude Encoding

We now explore amplitude encoding to encode *noun* vector representations in quantum circuits representing sentence meaning. The performance of quantum machine learning models, in which classical data are encoded onto quantum circuits, is compared to the models trained in Section 3 with regard to their training convergence and their predication accuracy. In Section 5, amplitude encoding is used to introduce classical information in the learning process of a VQC to reason about the connection between entropy and ambiguity.

The idea of amplitude encoding is to provide fixed parameters to the model to encode one particular quantum state on the qubit, instead of the model learning the noun parameters. This process might be advantageous because the model’s search space decreases in size. Furthermore, the initialisation and restriction of the parameters, making them static values, might be beneficial, because the model is provided the “correct” solution instead of learning it. Additionally, the search space becomes classically interpretable, which is the main reason why amplitude encoding is investigated in the current work (Section 5).

As for the disadvantages of amplitude encoding, it is unknown whether the particular vector embeddings are advantageous representations of the noun meanings because the representations learned by the model itself are unknown. Furthermore, the more parameters a machine learning model has, the better its ability to find complex functions connecting input and output data. By amplitude encoding, our quantum machine learning model’s parameters are restricted, which might limit this ability.

We restrict ourselves to the one-qubit approach in this section. This approach is justified by the fact that the cost of training a quantum machine learning model scales exponentially with the number of qubits used. For example, it takes significantly more resources to simulate quantum circuits encoding more complex sentences, such as

Person cooks meal and chef cooks meal.

In a non-normalised DisCoCat diagram, using the approach of encoding a noun onto one qubit, one would need 13 qubits to encode this sentence. While normalising the diagram minimises the number of qubits used (to six qubits), the resulting quantum circuit is still difficult to simulate classically.

More qubits provide a higher-dimensional Hilbert space to encode word meanings. In general, the number of qubits $n_{q}$ necessary to encode *d*-dimensional data onto a Hilbert space spanned by $n_{q}$ qubits is

$$n_{q}=\log_{2}\left(d\right)$$

### 4.1. Pre-Study

As a first investigation, we extract the states representing nouns by evaluating the corresponding sub-circuit in the learned model (from Section 3), which is as follows (using the IQP-ansatz): 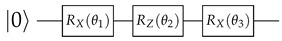

The resulting states are displayed on the Bloch sphere to investigate if the model has learned to map nouns belonging to different categories to similar sides on the Bloch sphere. Following research in quantum machine learning (Schuld et al. [56], Schuld and Killoran [57]), the learning process of a quantum machine learning model on a binary classification task entails maximising the distance of states, representing different categories, on the Bloch sphere. In our case, one might expect nouns with similar meanings (belonging to the same category, e.g., meal and sauce) to be mapped to similar vectors on the Bloch sphere, whereas nouns with opposite meanings (belonging to different categories, e.g., sauce and program) would be mapped to opposite sides. However, the quantum machine learning model does not learn a mapping from the nouns to the categories food or IT. Rather, it learns a mapping from combinations of nouns, adjectives, and verbs to the quantum states representing the sentences. Thus, while we expect the *sentences* to be mapped to opposite sides of the Bloch sphere (|0〉 and |1〉 by definition), our expectations toward the mapping of the *noun states* are less clear. We expect to see a categorising effect with respect to the position of the noun states on the Bloch sphere, as the model learns to differentiate between these vectors. Consider the two example sentences

person prepares meal.person prepares dinner.

The two sentences, only differing in the objects meal and dinner, are mapped to the same category, while a sentence in which, e.g., the object program is used, is mapped to the opposite category. For this reason, we expect there to exist a potentially weak correlation between the categories words belong to and their distance to each other on the Bloch sphere.

#### 4.1.1. Implementation

In total, there are nine nouns in the original dataset,

person, man, woman, dinner, meal, sauce, program, application, software

and nine nouns in the new dataset, where the words man and woman are replaced with the words chef and programmer. For the case of the two-dimensional encodings (onto one qubit), we discuss the encoding learned by the model using the Bloch sphere representation of the learned embeddings. We present the encodings for the three models: Tket, NumPy, and pennylane.

**Figure 10:**
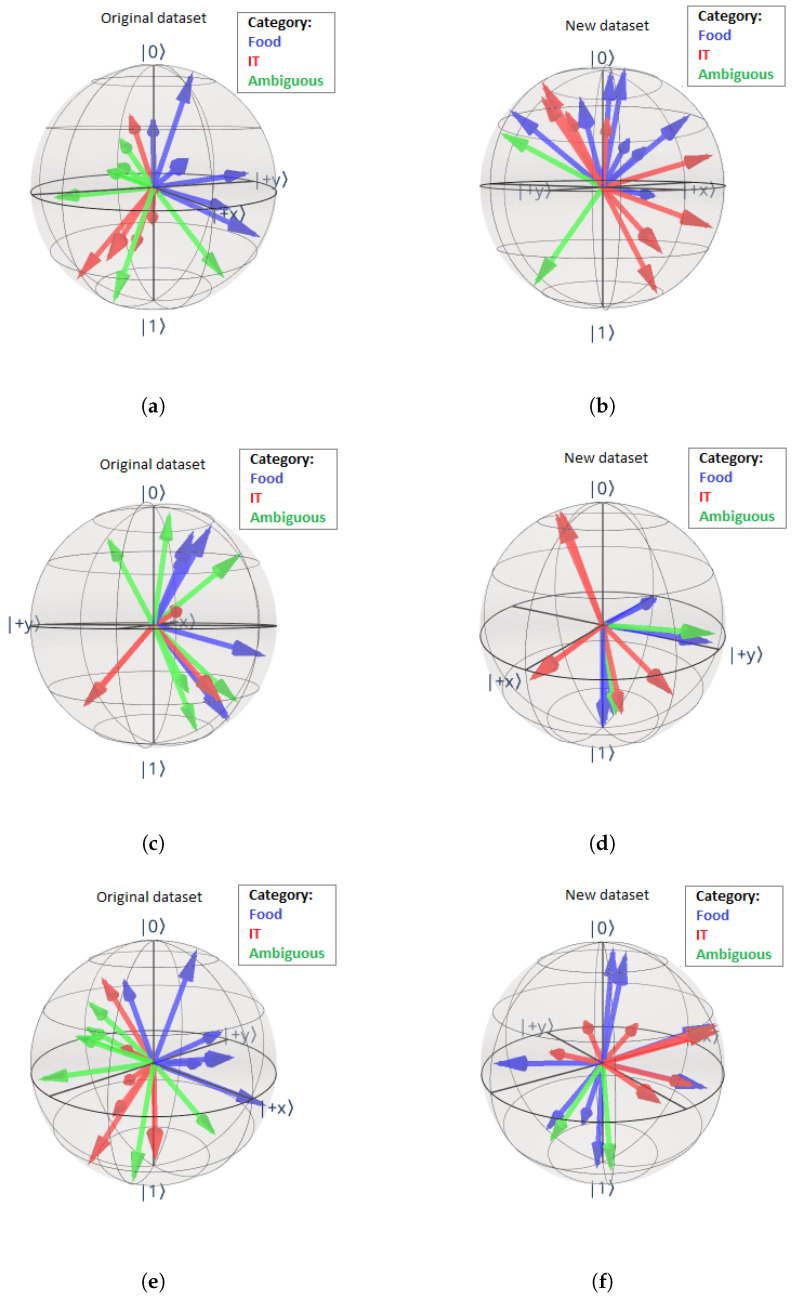
The nouns plotted on the Bloch sphere for both datasets. The blue colour indicates food, the red colour indicates IT, and the green colour indicates ambiguity between those two; as, e.g., in person for the Tket model (**a**,**b**), the pennylane model (**c**,**d**), and the NumPy model (**e**,**f**). All models are trained on the original dataset (**left column**) and new dataset (**right column**).

The noun states are shown on the Bloch sphere for the Tket model, both using the original dataset (Figure 10a) and the new dataset (Figure 10b). For the original dataset, we see a tendency of nouns with different meanings being encoded on similar parts of the Bloch sphere. The plot suggests that, while there is significant dispersion, nouns belonging to the category food (blue) are encoded into the subspace in which the *x*-, *y*-, and *z*-values (the *x*-, *y*-, and *z*-axes represent the axis labels in the 3-dimensional coordinate system in which the Bloch sphere is represented) are positive, whereas nouns belonging to the category IT (red) are encoded to state vectors for which the y- and z-values are negative, while ambiguous words (green) are encoded rather close to the nouns in the category IT. These interpretations are subject to high dispersion in the encoding, thus the results are limited in their explanatory power. The observations for the two different datasets vary strongly. Using the new dataset, nouns in the two different categories are not encoded to different sides of the Bloch sphere, while some ambiguous words are encoded to entirely different states. This means that the encoding mechanisms that the model learns do not necessarily encode words belonging to opposite states on opposite sides of the Bloch sphere.

Figure 10c,d depict the noun states for the pennylane model for both datasets. The pennylane model does not coherently encode words of the same category to the same side of the Bloch sphere for either of the datasets. The encoding obtained from the NumPy model (Figure 10e,f) is very similar to the encoding resulting from the Tket model. For the original dataset (Figure 10e), there is a pattern present; namely, words belonging to the categories food and IT are encoded to different sides of the Bloch sphere, and ambiguous words are encoded closer to the nouns in the category IT. The encodings of the nouns in the new dataset do not follow a pattern, where the dispersion of the vectors is even more prominent than for the Tket model (Figure 10f). So far, the NumPy and Tket models are performing very similarly on the investigated tasks.

##### Discussion and Further Investigation

The figures shown above are not averaged. This is because when we retrain the model, the VQC gate parameters are initialised randomly. Thus, we obtain entirely different mappings for the individual nouns. To obtain an average value for the positions of the nouns relative to one another, additional metrics must be introduced, such as the distances between nouns of different categories. These measures may then be averaged across multiple runs.

It is beyond our intuition why the encoding of the noun states for the Tket model trained on the new dataset differs from the encoding of the model trained on the original dataset so substantially. When comparing the encodings of nouns in the original and new datasets, the latter (right column in Figure 10) indicates that the positions on the Bloch sphere of different nouns relative to each other are not correlated with the noun meanings in the way we expected. However, there is a certain correlation between noun meaning and the position of the state vector representation on the Bloch sphere, as we can see for the original dataset (left column in Figure 10). We address these plots again in Section 4.2.1.

The pennylane model, which is the best-performing model in Section 3.2, learns parameters in an abstract way that is contrary to our expectations. However, the fact that the model is performing best does not necessarily imply that the relations learned by the model are the most intuitive, or suitable for reasoning with.

We notice one point that applies to all the models. When mapping sentences to quantum circuits, upon normalisation some of the states representing words in the sentence are daggered (†) or turned upside down in the diagrams. Thus, because the same word occurs in different sentences, there are quantum circuits in which the word is in a daggered state, and others in which it is in a non-daggered state. In the lambeq training pipeline, the daggered and non-daggered representations have different parameters assigned to them. This means that these two representations are learned as though they are different words and not different representations of the same word. This is why, in the Bloch sphere figures above, each word’s state is depicted twice (i.e., we count twice the amount of arrows compared to the amount of nouns we have available in each category for the respective datasets for the NumPy and Tket models). Ultimately, this means that if the Bloch sphere contains more distinct vectors than the number of nouns in the particular dataset for the specific category, different representations for the same word are not encoded to the same state on the Bloch sphere. If this happens, one possible explanation is that it is due to the different representations (daggered and non-daggered) occurring in slightly different contexts in the sentences. Furthermore, an important effect on the model’s performance is the limited amount of data available for training.

The pennylane model is the only model that learns daggered and non-daggered representations of words to the same state for both datasets (Figure 10c,d). However, with an exception: the different representations for each of the *ambiguous* words are encoded to different parts of the Bloch sphere for both datasets. We argue that this makes sense since non-ambiguous words are unlikely to occur in entirely different contexts (e.g., the word meal is more likely to appear in connection with other words belonging to the category food, like cooks or delicious), whereas ambiguous words can appear in entirely different contexts (e.g., the word person could appear either in connection with the word meal or program). Thus, the learned states for the different representations of ambiguous words (Eperson) are more likely to be different to each other than those for non-ambiguous words (e.g., meal).

**Figure 11:**
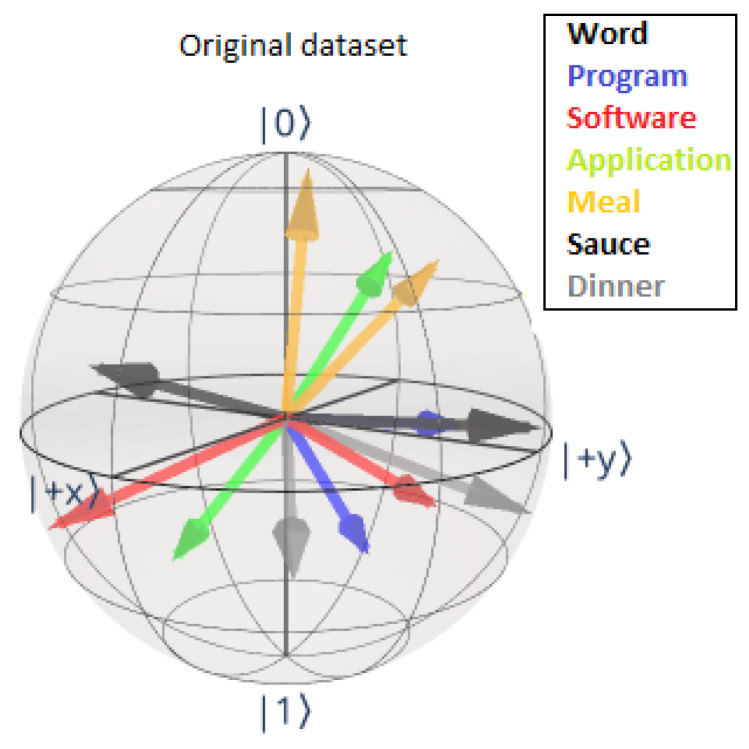
The states that the individual words are mapped to in the learning process of the model using the Tket model trained on the original dataset. Both representations are shown, the daggered one and the non-daggered one, for each word. For an explanation, see the main text.

We clarify the above by focussing on the explicit encodings of the nouns in the categories food and IT by the Tket model using the original dataset. The corresponding noun encodings on the Bloch sphere are shown in Figure 11. We see that, for most words, the states of the undaggered and daggered states are considerably far apart, emphasising the inherent error present in the model.

In conclusion, for both the NumPy and the Tket models, we see that for the original dataset words of similar meaning are mapped to only approximately similar places on the Bloch sphere. For the new dataset, there is no correlation between the similarity of word meanings and the mapping onto the Bloch sphere. This difference in encoding regarding the original and the new datasets is relevant for the discussion of the model’s performance when amplitude encoding is used in Section 4.2.1. Given these observations, we hypothesise that amplitude encoding will have more advantageous effects on the model trained using the original dataset than on the model trained using the new dataset. While the encoding by the pennylane model varies from the Tket model, the encoding scheme resulting from the NumPy model is very similar to that of the Tket model. The sections to come only address the Tket model.

### 4.2. Encoding Data on One Qubit

When encoding data onto one qubit, nouns belonging to the category food are amplitude-encoded to the state |1〉, and nouns belonging to the category IT are amplitude-encoded to the state |0〉, so that they are encoded to opposite sides of the Bloch sphere.

#### 4.2.1. Implementation

**Figure 12:**
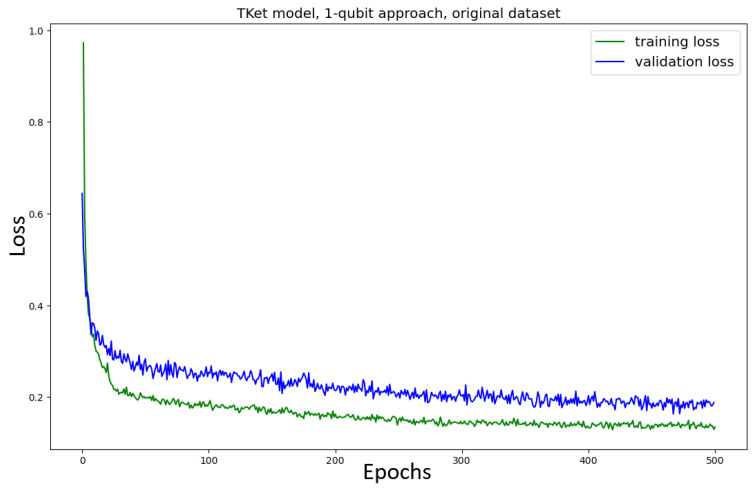
Tket model with noun meanings amplitude-encoded on one qubit for original dataset. Metrics: accuracy = 0.917, κ = 0.733, $F_{1}$-score = 0.857.

**Figure 13:**
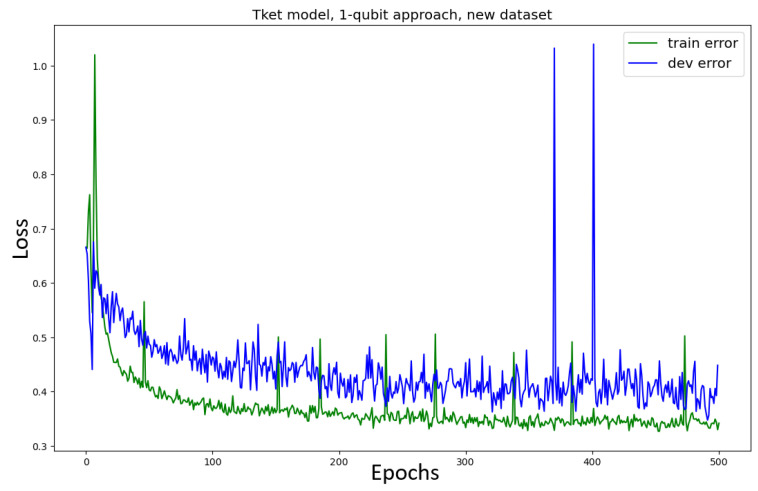
Tket model with noun meanings amplitude-encoded on one qubit for new dataset. Metrics: accuracy = 0.783, κ = 0.533, $F_{1}$-score = 0.696.

The Tket model is trained on the original dataset (Figure 12) and the new dataset (Figure 13). The loss curves for the pennylane and NumPy models, where amplitude encoding onto one qubit is used, are in the Appendix B, in Figure A12 (pennylane, original dataset), Figure A13 (pennylane, new dataset), Figure A11 (NumPy, original dataset), and Figure A14 (NumPy, new dataset). For the new dataset, there is a substantial drop in performance (≈23% for the accuracy, ≈62% in κ, and ≈34% in the $F_{1}$-score) compared to the non-amplitude-encoded model (Figure 8). This difference in performance between the models trained on the two different datasets did not occur in the non-amplitude-encoded case (Figure 7 and Figure 8).

Using amplitude encoding has no noticeable impact when training the model on the original dataset: while the amplitude-encoded model has a slightly higher accuracy (≈2%), it has a lower $F_{1}$-score (≈5%) than the non-amplitude-encoded model (Figure 7).

Discussion

The results gathered in this section suggest that restricting the parameters of the model while providing encodings of the nouns does not affect the performance of a model trained on the original dataset. However, it does affect the model’s performance when trained on the new dataset. This implies that the chosen embeddings are better suited for the model trained on the original dataset than for the model trained on the new dataset. We argue that this might be the result of amplitude-encoding both subjects and objects to opposite sides of the Bloch sphere in the new dataset. This is because, in the original dataset, the model had to learn mappings from superposition states to either pole of the Bloch sphere, where all the subjects are encoded to the same state on the Bloch sphere. However, in the case of the second dataset, the subjects are encoded to superposition states and, additionally, to the states |1〉 and |0〉. This means that the mapping to the sentence space learned by the model is a more complex one because the verb itself must be able to discriminate the input variables more clearly. This more complex representation of verbs leads to an overall more complex problem, which ultimately yields a lower convergence speed and accuracy.

Additionally, for the model’s encoding of vectors representing nouns on the Bloch sphere (Figure 10), the encodings for the new dataset (right side in Figure 10) are not as coherent as for the original dataset (left side in Figure 10), as mentioned in Section 4.1. If the model does not learn the representations that are amplitude-encoded, the approach itself of enforcing these encodings is likely flawed. The parameter search space is restricted, which limits the model’s ability to process the potentially suboptimally encoded state vectors representing noun meanings.

### 4.3. Encoding Data on Two Qubits

Motivated by Hoffmann [8], we follow the approach presented by Kerenidis and Prakash [58] to encode data on two qubits. Starting from a four-dimensional array *w*, it is put in a two-qubit quantum state as follows:

(28) $$|w\rangle =\frac{1}{\left|w\right|}\sum_{i=1}^{n=4}w_{i}|i\rangle$$

where |i〉 is the basis vectors of the four-dimensional Hilbert space spanned by two qubits, and $w_{i}$ is the classical vector *w* components. The encoding procedure works as a series of rotation and controlled rotation gates applied to two qubits.

**Figure 14:**
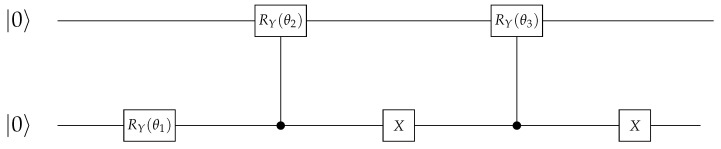
The quantum circuit used to amplitude-encode the meaning of a four-dimensional vector onto the Hilbert space spanned by two qubits. The parameters are chosen based on the components of the classical vector to be encoded [58].

The parameters of the corresponding circuit initialising these states are chosen to be combinations of the components of the classical vector *w* (Figure 14). This circuit replaces the unitaries encoding the meaning of the nouns in amplitude encoding.

A next important step is gathering the data that will be amplitude-encoded on the quantum circuits. There are numerous models trained on large collections of data to obtain vector representations for words (e.g., word2vec [59,60]).

Yamada et al. [61] trained a word2vec model on all of Wikipedia and called the resulting model Wikipedia2Vec. The high-dimensional datasets are available online (https://wikipedia2vec.github.io (accessed on 12 August 2024)), where the lowest dimensional vector space is 100-dimensional. We proceed by reducing the dimensionality of this vector space, following Hoffmann [8], to four dimensions. To reduce the dimensions of the given vector spaces, we use principal component analysis and independent component analysis.

#### 4.3.1. Principal Component Analysis and Independent Component Analysis

Principal component analysis (PCA) [62] reduces the number of dimensions in a given dataset using the concept of principal component vectors (*principal components*). These principal components are uncorrelated and chosen in such a way that they carry a maximal amount of the initial information in the dataset. Principal components are eigenvectors of the *covariance matrix* [63]. The eigenvectors with the highest corresponding eigenvalues indicate in which direction (for which principal component) the variance is maximal. The principal components explain a certain part of the initial variance in the dataset. Depending on how many of these components there are, the initial variance explained by the set of principal components varies. The *explained variance*, i.e., the ratio between the variance that is explained by a number of principal components and the variance in the original data, is a suitable measure for how much information is lost in the process of reducing the dimensionality of a dataset using PCA.

**Figure 15:**
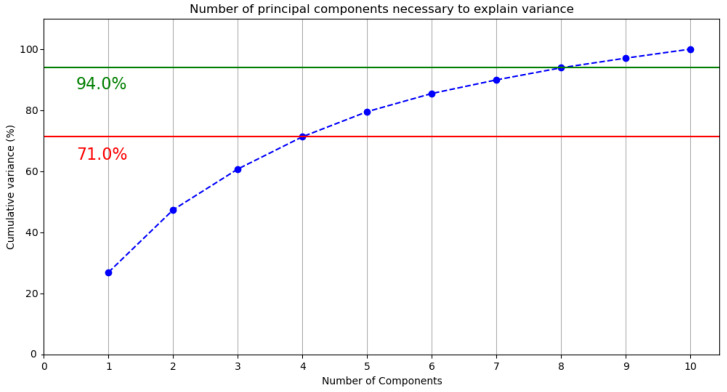
Cumulative variance explained by PCA for a varying number of principal components. This result justifies the reduction of the 100-dimensional Wikipedia2Vec space to four principal components.

The evolution of the cumulative variance, i.e., the sum over the variance of each principal component, with the number of principal components, is displayed in Figure 15 for the 100-dimensional Wikipedia2Vec dataset. The code for creating this plot is available online (https://cinnipatel.medium.com/principal-component-analysis-python-a6214346cae7 (accessed 12 August 2024)). Four components explain 71% of the initial variance (red line in Figure 15). Upgrading to one more qubit, and thus four more dimensions, would yield 94% of the initial variance (green line in Figure 15) being explained by the eight involved principal components. We also note that reducing the dataset to two dimensions yields a loss in the initial variance of over 50%. The cumulative variance converges to 100% as more principal components are added (Figure 15).

As a second method, *independent component analysis* (ICA) [64] is applied to reduce the dimensionality of the Wikipedia2Vec dataset. In ICA, the components are statistically independent of each other. In contrast to PCA, the ICA algorithm tries to find vectors that are independent components of the data at hand. ICA maximises the extent to which these components are statistically independent of each other. Both PCA and ICA are applied and the ultimate performances are compared to each other.

Before applying PCA and ICA to the Wikipedia2Vec dataset, the data were scaled between 0 and 1 using the MinMaxScaler method of the python library sklearn (https://scikit-learn.org/stable/ (accessed on 12 August 2024)).

#### 4.3.2. Implementation—PCA

**Figure A9:**
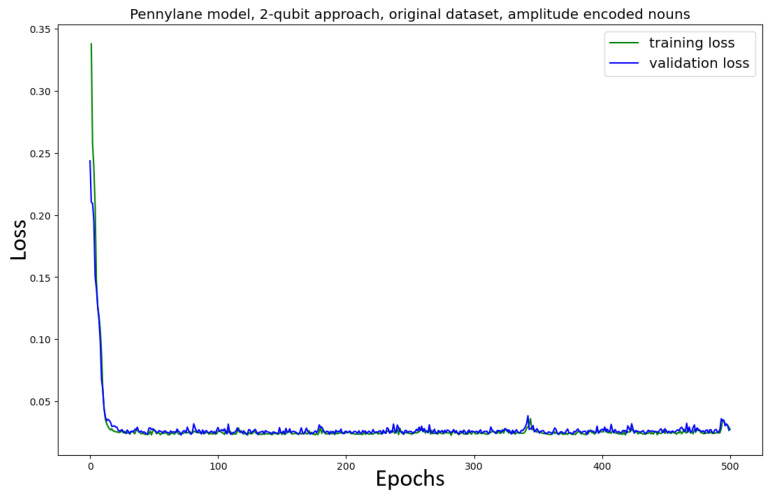
pennylane model, trained over 500 epochs, amplitude-encoded onto two qubits using PCA on the original dataset. Metrics: accuracy = 1.0; κ = 1.0; F_1_-score = 1.0.

**Figure A10:**
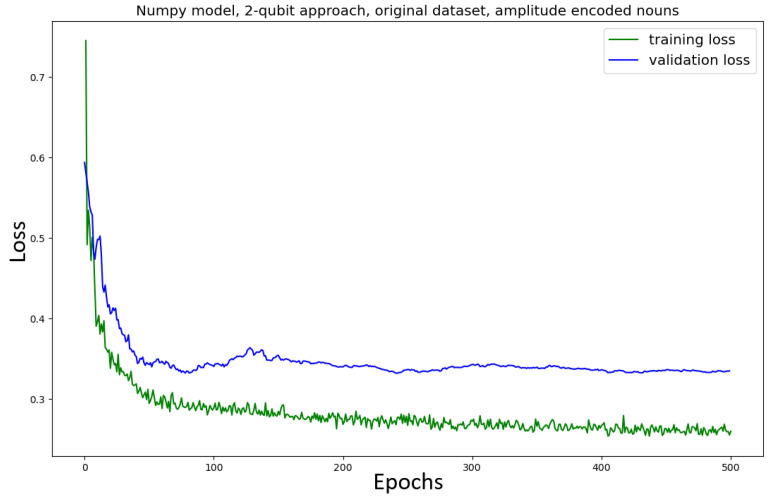
NumPy model, trained over 500 epochs, amplitude-encoded onto two qubits using PCA on the original dataset. Metrics: accuracy = 0.93; κ = 0.87; F_1_-score = 0.93.

**Figure A11:**
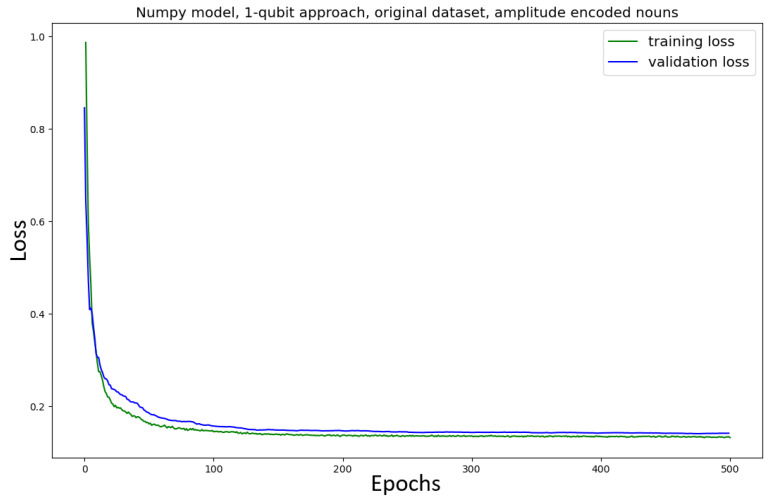
NumPy model, trained over 500 epochs, amplitude-encoded onto one qubit on the original dataset. Metrics: accuracy = 0.97; κ = 0.87; F_1_-score = 0.94.

**Figure A12:**
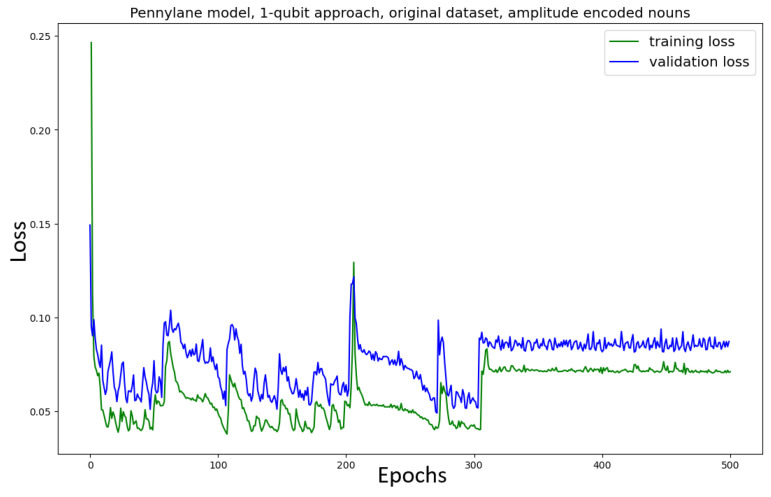
pennylane model, trained over 500 epochs, amplitude-encoded onto one qubit on the original dataset. Metrics: accuracy = 0.93; κ = 1.0; F_1_-score = 1.0.

**Figure A13:**
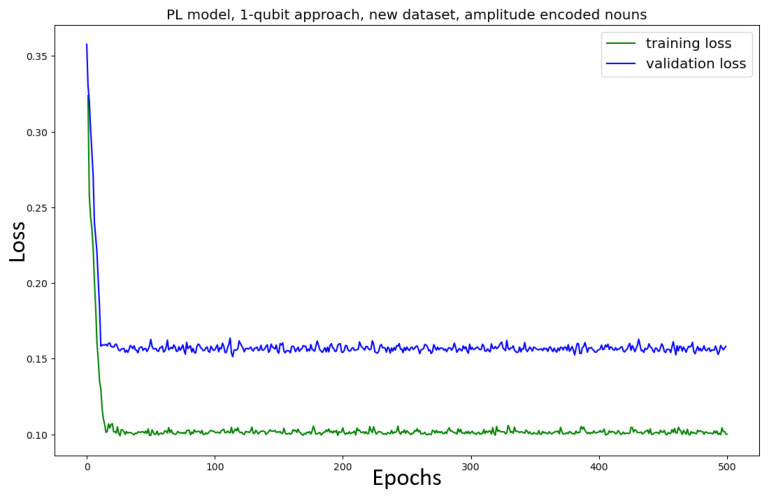
pennylane model, trained over 500 epochs, amplitude-encoded onto one qubit on the new dataset. Metrics: accuracy = 0.77; κ = 0.53; F_1_-score = 0.70.

**Figure A14:**
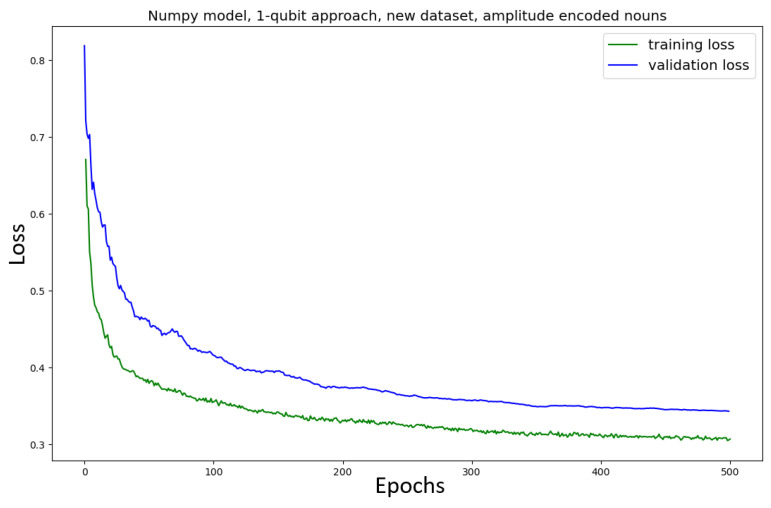
NumPy model, trained over 500 epochs, amplitude-encoded onto one qubit on the new dataset. Metrics: accuracy = 0.80; κ = 0.60; F_1_-score = 0.75.

**Figure A15:**
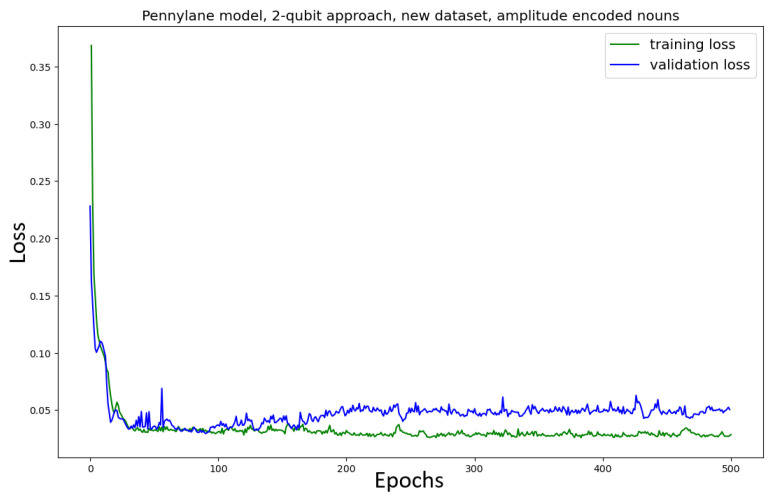
pennylane model, trained over 500 epochs, amplitude-encoded onto two qubits using PCA on the new dataset. Metrics: accuracy = 0.93; κ = 0.88; F_1_-score= 0.90.

**Figure A16:**
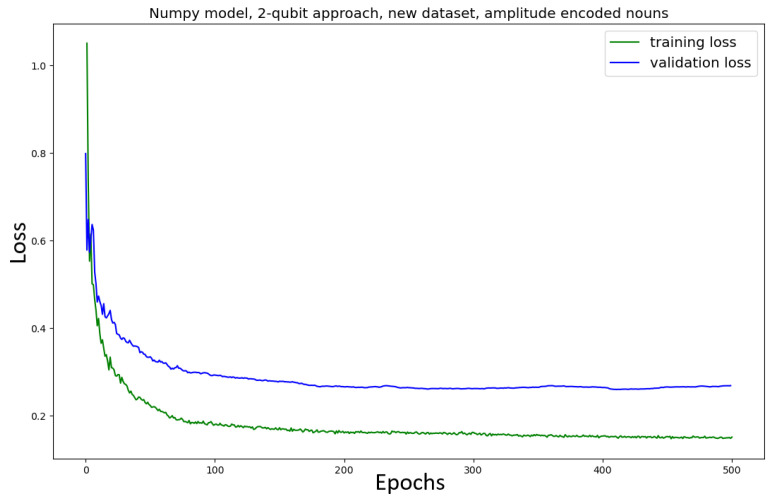
NumPy model, trained over 500 epochs, amplitude-encoded onto two qubits using PCA on the new dataset. Metrics: accuracy = 0.97; κ = 0.87; F_1_-score = 0.94.

In this section, PCA is used for the dimensionality reduction of the classically trained vector spaces. The loss curves for the pennylane and NumPy model, where the noun meaning is encoded onto two qubits and PCA is used for the dimensionality reduction, are shown in the Appendix B, Figure A9 (pennylane, original dataset), Figure A15 (pennylane, new dataset), Figure A10 (NumPy, original dataset), and Figure A16 (NumPy, new dataset).

**Figure 16:**
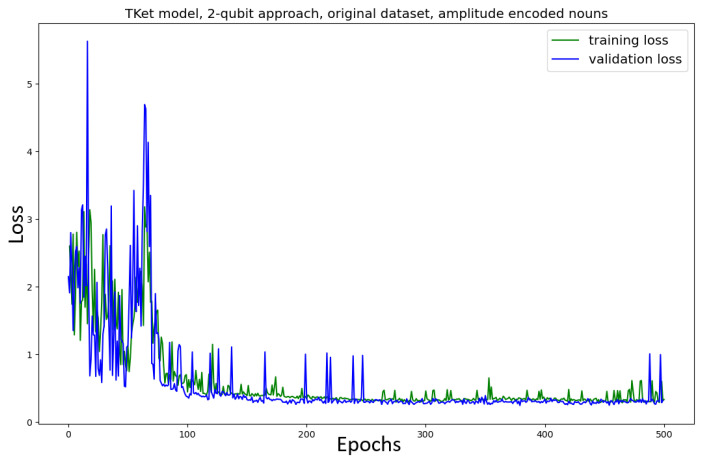
Convergence of the Tket model using the two-qubit approach, with the nouns amplitude-encoded according to the Wikipedia2Vec vector space, using the original dataset, and using PCA. Metrics: accuracy = 0.900; κ = 0.733; $F_{1}$-score = 0.857.

In Figure 16, the loss curve for the case in which we use PCA to reduce the dimensions is depicted for the original dataset. The convergence of this model is substantially faster than in the non-amplitude-encoded case (Figure 9, top). While the model (Figure 9, top) does not fully converge, the current model (Figure 16) converges after 100 epochs. Furthermore, the accuracy in the amplitude-encoded case is (≈15%) higher, as compared to the non-amplitude-encoded model (Figure 9, top). The κ- and the $F_{1}$-scores are, however, slightly lower for the amplitude-encoded case (≈9%).

**Figure 17:**
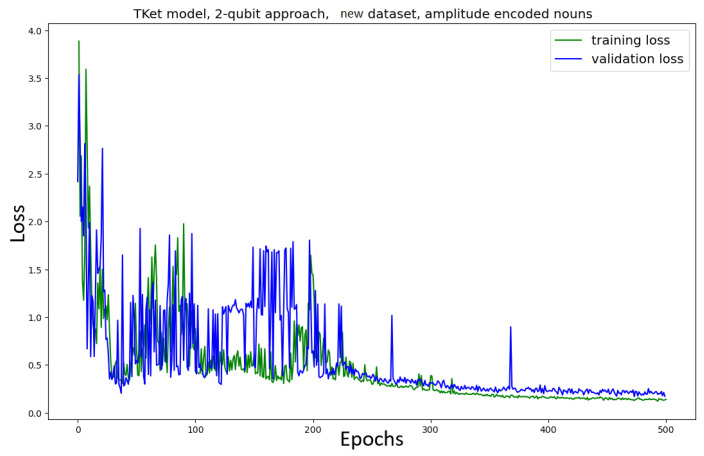
Convergence of the Tket model using the two-qubit approach, with the nouns amplitude-encoded according to the Wikipedia2Vec vector space, using the new dataset, and using PCA. Metrics: accuracy = 0.890; κ = 0.851; $F_{1}$-score = 0.815.

The model is trained on the new dataset (Figure 17). When training the model on the new dataset, the model converges after around 250 epochs (Figure 17). This is in contrast to the non-amplitude-encoded approach (Figure 9, bottom), which shows no clear convergence. All metrics are better for the amplitude-encoded case than the non-amplitude-encoded case (≈8% across the metrics). The model trained on the new dataset takes 150 epochs longer to converge, but the metrics in the two cases are very similar to each other.

Discussion

The results for the model trained on the new dataset suggest that amplitude-encoding nouns onto two qubits increases the model’s ability to converge. The results for the model trained on the new dataset support the hypothesis that amplitude encoding leads to faster convergence of the model, as well as the hypothesis that it increases overall performance. There is a substantial difference between the impact of amplitude encoding on one and two qubits. Our results suggest that amplitude encoding has an advantageous impact when the meaning of a noun is encoded onto two qubits. However, for the one-qubit approach, there was no noticeable effect on a model trained on the original dataset (Figure 12), while the overall performance of a model trained on the new dataset (Figure 13) was decreased.

There could be multiple reasons for this difference in performance when amplitude-encoding onto one or two qubits. Firstly, the two-qubit approach has a higher number of parameters. This means that restricting a certain number of parameters does not impact the model’s search space as much as in the one-qubit approach. Secondly, the vector embeddings for the nouns might be more suited for the model than they are in the one-qubit approach. In the two-qubit approach, actual data trained from Wikipedia is used, while in the one-qubit approach, nouns belonging to different categories are assigned opposite vectors on the Bloch sphere, which might be a suboptimal encoding for the model.

In the two-qubit approach, the process of restricting the parameters of the model alone might increase the model’s performance by limiting the complexity of the search space. There exists an important trade-off between *exploitation* and *exploration*. Generally, by adding parameters to the model, it can learn more intricate relations between input and output data. However, since the search space grows with the number of parameters, the model is more likely to converge to a suboptimal solution. In a bigger search space, there are more parameters to *explore*, but it becomes harder to choose a solution to *exploit*. Therefore, it might be that it is not mainly the quality of our embedding choice for the individual vectors that makes the model perform better, but rather the process of restricting the parameters.

#### 4.3.3. Implementation—ICA

**Figure 18:**
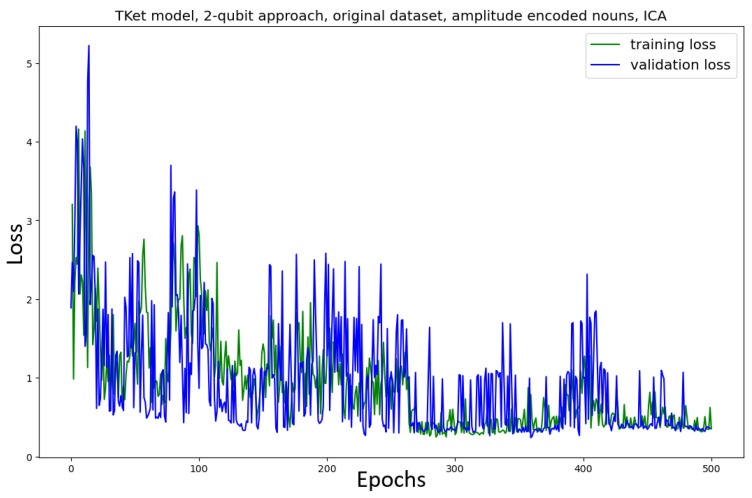
Convergence of the Tket model using the two-qubit approach, with the nouns amplitude-encoded according to the Wikipedia2Vec vector space using ICA on the original dataset. Metrics: accuracy = 0.767; κ = 0.600; $F_{1}$-score = 0.769.

**Figure 19:**
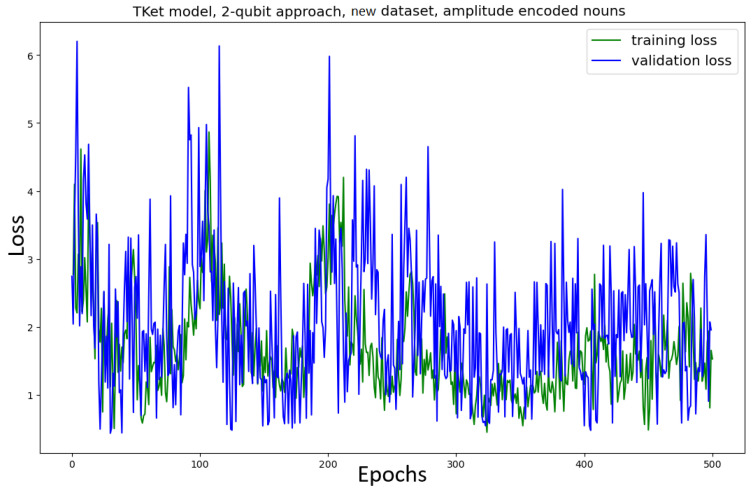
Convergence of the Tket model using the two-qubit approach, with the nouns amplitude-encoded according to the Wikipedia2Vec vector space using ICA on the new dataset. Metrics: accuracy = 0.633; κ = 0.500; $F_{1}$-score = 0.692.

In light of this argument, ICA instead of PCA is considered as a process of dimensionality reduction. In this, we vary the embeddings themselves and hope to investigate the impact that the embedding choice has on the performance of the model (Figure 18 for the original dataset; Figure 19 for the new dataset). For the model using the original dataset, the convergence is significantly slower. Furthermore, the performance metrics are significantly worse (≈15% less accuracy, ≈18% lower κ, ≈10% lower $F_{1}$-score) as compared to the PCA approach (Figure 16). The model using the new dataset does not converge, and its performance metrics are substantially worse than the approach using PCA (Figure 17) (≈29% less accuracy, ≈41% lower κ, ≈15% lower $F_{1}$-score).

For both models, trained on the original and new datasets, the convergence of the amplitude-encoding approach using ICA is faster than the approach without amplitude encoding (Figure 9). However, the performance resulting from the ICA approach is substantially worse than the performance of the non-amplitude-encoded two-qubit approach model (Figure 9. For the original dataset, the performance is ≈15% worse across the metrics; for the new dataset it is ≈25% worse across the metrics).

Discussion

Firstly, we note the faster convergence in the PCA approach, yet lower overall performance of the model trained on the new dataset. This implies that the restriction of parameters by amplitude encoding helps the model converge. Secondly, our results suggest that the embeddings given by the ICA approach are suboptimal for both the models trained on the original and the new datasets. Using ICA as a method to obtain vector embeddings for amplitude-encoded nouns leads to worse performance than using PCA, which indicates that the choice of embedding impacts the model’s performance. As stated in the introduction to PCA and ICA in Section 4.3.2, PCA seeks to find components in the data explaining a maximal amount of variance of the original dataset, while ICA seeks to maximise the independence of the individual components. ICA is preferred when the data are a mixture of individual sources, e.g., in images or audio signals [64], while PCA is the preferred method to obtain a lower-rank representation of a given dataset [62], which makes PCA more suited for the task at hand.

We argue that the process of restricting the model’s parameters is not the only reason for the advantageous effect amplitude encoding has on the performance of models in the two-qubit approach. The results suggest that the process of amplitude encoding is effective for the two-qubit approach. While the process of restricting the parameters of the model (independent of the choice of vector embedding) still has a considerable effect on the model’s convergence, the choice of embedding has a substantial impact on the performance. This means that, based on our results, not only does amplitude encoding reduce the dimensions of the search space by restricting parameters, but it also initialises the remaining parameters in an advantageous way, helping the model converge and ultimately perform better. The reasons we see this more prominently in the two-qubit approach are twofold. First, the search space in this case is higher-dimensional and therefore the reduction in dimensionality has a higher impact. Second, two qubits are able to capture more linguistic information about the underlying word meaning. The encoding of the words may thus play a more crucial role for the two-qubit approach.

## 5. Entropy, Fidelity, and Ambiguity

A density matrix can represent the meaning of a word, a sentence, or any other linguistic entity. In this section, we address the connection between the entropy of a density matrix and the linguistic ambiguity that it represents in the sentence. In particular, we discuss the link between variations in the entropy of a density matrix and variations in the linguistic ambiguity of the sentence it represents, and the effect that amplitude encoding has on this connection. The motivation behind this approach is the following. The more ambiguous a sentence is concerning its category, the less certain the model is about what category it belongs to. As a result, the probability distribution returned by the model is more evenly distributed, which yields a higher value of the entropy. Thus, the ambiguity in a sentence is expected to be correlated with the entropy of the density matrix that represents this sentence. In addition to entropy, fidelity is used as a measure of how ambiguous a sentence represented by some density matrix is. While the entropy of a quantum probability distribution will be connected to the ambiguity in a sentence, the fidelity indicates the connection between individual probabilities in the distribution and the categories. We restrict ourselves to the one-qubit approach.

### 5.1. Approach

To form ambiguous sentences from the datasets, we forget words in sentences (Section 2.6). After having forgotten words, disambiguating words are added to the ambiguous sentences. With this procedure, the level of ambiguity in sentences is varied and entropy and fidelity can be recorded for varying ambiguity levels.

Two different ways of forgetting nouns have to be distinguished. The forgetting of a daggered noun is diagrammatically captured by Equation (29).(29)
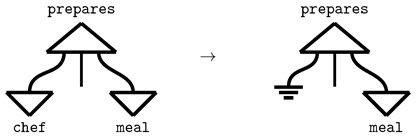

The noun qubit is simply discarded in this case. Forgetting a non-daggered noun in a sentence is captured by Equation (30).(30)
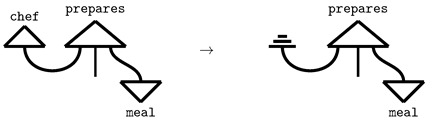

In this case, the noun is replaced with the maximally mixed (Bell-)state. We forget both subjects and objects in sentences (but never both subject and object in the same sentence). Note that by one word in the diagram being represented by a (maximally) mixed state, the meaning of the whole sentence is represented by a density matrix representing a mixed state. This means that what is depicted in Equations (30) and (29) is a probability distribution over diagrams, which each represent a pure state, where the pure states correspond to all possible realisations of the sentence with respect to the discarded word.

In the original dataset, all subjects are ambiguous:

man, woman, person

The sentences’ objects, on the other hand,

meal, dinner, sauce, code, program, software

are not ambiguous. For the original dataset, we hypothesise that forgetting the object in the sentence will be correlated with more uncertainty, and thus a higher entropy of the resulting density matrix, than forgetting the subject. This is because, if the object remains, the sentence can be still classified into a category. However, if the object is missing and only an ambiguous subject remains, the sentence cannot be classified into either category any more.

The new dataset introduces the non-ambiguous subjects chef and programmer. We still, for the new dataset, expect the entropy for the case of the subject being forgotten to be lower. However, since there is only one ambiguous subject (person), we expect this relation to be less prominent compared to in the original dataset.

### 5.2. Disambiguation

It is possible to introduce further information to the sentence by adding words. We introduce three sentence types. For these three sentence types, the entropy and ambiguity are recorded in Section 5.4. Sentence type 1 is

Sentence Type 1. subject prepares object.

Note that the verb prepares is the only ambiguous verb, which is why we only use this verb here. To this, we add a phrase to obtain sentence type 2:

Sentence Type 2. subject prepares object and verb* it.

In both datasets, verb* is one of the following:

cooks, bakes, debugs, runs

**Figure 20:**
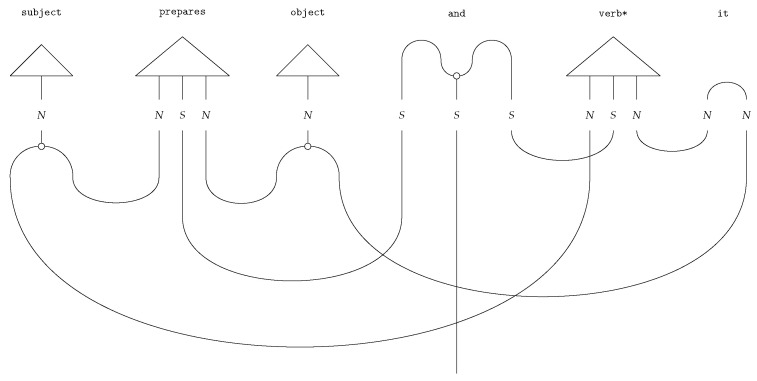
The DisCoCat diagram encoding the meaning of the sentence subject prepares object and verb* it.

**Figure 21:**
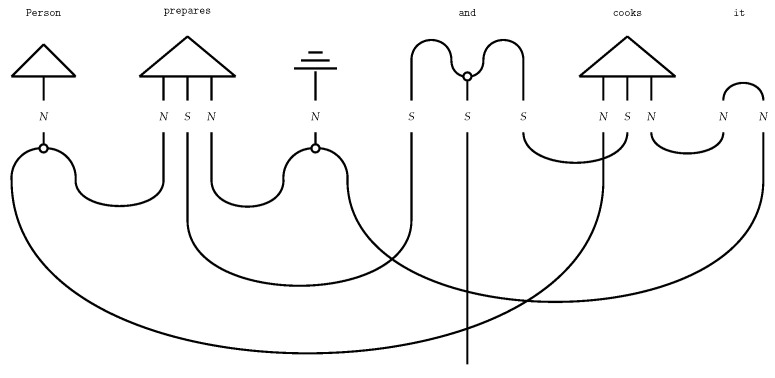
The diagram encoding the sentence subject prepares … and verb* it, where the object is replaced with the completely mixed state.

While for sentence type 1, the DisCoCat diagram is straightforward, for sentence type 2, the DisCoCat diagram is more involved (Figure 20) and can be mapped to a quantum circuit. Note that the words it and and are not in the dataset, but they are displayed in the above diagram entirely in terms of wires. Thus, we will model sentences of both the first and second kind using quantum circuits arising from the presented DisCoCat diagram. Upon discarding noun qubits in the circuit (either corresponding to person or meal in the example above), we introduce linguistic ambiguity, which we then connect to the entropy of the density matrices representing the sentences. An example diagram in which the word meal is forgotten is depicted in Figure 21, where thick wires indicate density matrices and completely positive maps. The derivation of this diagram, using Lambek calculus with extensions, following Wijnholds [14], is displayed in Appendix A. This type of disambiguation is possible with both the new and the original datasets.

Additionally, due to the existence of non-ambiguous subjects in the new dataset, we introduce sentence type 3:

Sentence Type 3. subject prepares object and subject* does too.

where either subject or object are forgotten. In the above example, subject* is either chef or programmer. The DisCoCat diagram for the above example, following Wijnholds [14], is where the object in the sentence is forgotten. This structure is mapped to a quantum circuit to investigate the entropy of the density matrices representing these sentences.

### 5.3. Amplitude Encoding

In the case of *amplitude-encoded* nouns, meaning is encoded to either of the vectors |0〉 or |1〉.

To understand the state of the discarded qubit, we remember that the original task is the classification into either the food or the it category. All sentence meanings evaluated exist with respect to the binary classification task that the models were originally trained on. This means that all density matrices represent probability distributions over predictions as to what category the sentence modelled by the density matrix belongs to. To understand the state of the discarded qubit, remember the original labels food=|1〉,IT=|0〉. From these pure states, we construct the density matrices representing the pure states $\rho_{\mathrm{food}}$ and $\rho_{\mathrm{IT}}$ in Equation (31).

(31) $$\rho_{\mathrm{food}}=|0\rangle \langle 0|=\left(\begin{array}{cc} 1 & 0\\ 0 & 0 \end{array}\right)\;\;\rho_{\mathrm{IT}}=|1\rangle \langle 1|=\left(\begin{array}{cc} 0 & 0\\ 0 & 1 \end{array}\right)$$

The discarded qubit thus represents an equal probability distribution over the two categories:

(32) $$\rho_{\mathrm{discarded}}=\frac{1}{2}\;\cdot \;\left(\begin{array}{cc} 1 & 0\\ 0 & 1 \end{array}\right)=\frac{1}{2}\;\cdot \;\left(\rho_{\mathrm{food}}+\rho_{\mathrm{IT}}\right)$$

In the case of the model *learning* the noun parameters in the training procedure, the nouns do not correspond exactly to the states of either |0〉 or |1〉 but instead are represented by superpositions over these two states. Furthermore, the nouns in the category food will not necessarily be opposite to the nouns in the category IT (Section 4.1). This means that when amplitude encoding is not applied, the discarded qubit does not necessarily describe an equally weighted probability distribution over the two categories. As a consequence, we expect the models in which the noun meaning is amplitude-encoded to perform better in the experiment (Section 5.4).

### 5.4. Implementation

We investigate the effect of amplitude encoding on the relation between linguistic ambiguity and mixedness of density matrix representations.

Concretely, both the *von Neumann entropy* (Equation (22)) and the *fidelity* (Equation (23)) are applied as measures of how mixed a prediction by the model is. The fidelity as a measure is employed as the comparison of the predicted density matrix with the correct density matrix (corresponding to either of the two categories used). This means that optimally the fidelity would be 1, whereas a fidelity of 0 would imply no similarity at all between the correct and predicted categories. We explain this in more detail below.

The experiment is constructed as follows. First, all possible sentences that can be composed for each of the three sentence types using the two datasets are considered. For sentence type 1, there are three subjects and six objects available, which means there are 18 sentences of this type available. For the new dataset, we only use ambiguous subjects. We do not consider sentences containing, e.g., the word chef as the only subject. Firstly, this allows for comparison of the performances of the models trained on the two different datasets; and secondly, in sentences of type 3, the nouns chef and programmer are used for disambiguation. As a result, there are only six sentences that we consider for the second dataset.

Similar to sentences of type 2, for each of the sentences of type 1 we have two possible phrases to extend the sentences with. There are four verbs:

cooks, bakes, debugs, runs

Every sentence of type 1 can only be meaningfully extended by two of these verbs. This means that, in this sentence type, we can build 36 distinct sentences for the original dataset and 12 sentences for the new dataset. When interpreting the results in Section 5.4, we have to keep in mind that for the original dataset, when investigating sentences of type 1 and type 2, the average is taken over three times more sentences as compared to the new dataset. As a consequence, the results for the models trained on the original dataset might be more accurate than for the models trained on the new dataset.

Lastly, for sentences of type 3, there are six sentences that we can construct with the vocabulary only of the new dataset, as reasoned above. This is because we only have the two words chef and programmer at our disposal.

The next step is to forget words in the sentences. As in this study, we restrict ourselves to manipulating the nouns; for each of the sentences of the three sentence types (1, 2, and 3), we forget either the subject or the object of the sentence. For each sentence for the respective sentence type, we obtain two density matrices with either the subject or object forgotten.

The results discussed in the following section report values of average entropy and average fidelity. Firstly, the entropy of each density matrix is determined and the average over these values is taken. For example, for sentences in which the object is forgotten, the entropy of all density matrices representing quantum circuits in which the object qubit is discarded is considered and averaged. For the fidelity values, the process is slightly more involved.

We use the fidelity to determine how similar the above-determined density matrices, modelling predictions corresponding to sentences in which either subject or object are forgotten, are to the density matrices representing the pure states in Equation (31). Then, similarly to the entropy above, we average over all realisations of either object or subject being forgotten in a given sentence. By using fidelity as a measure, it is possible to not only assess if the ambiguity is being lowered (as with the entropy), which amounts to the model being more certain about its prediction, we can furthermore reason about the model’s ability to make correct predictions.

Note that all entropy values discussed in this paper are determined using base 2 of the logarithm, which results in the maximum entropy value being 1, rather than ln(2) when choosing base *e* for the logarithm (as is the case in the definition of the von Neumann entropy in Equation (22): the maximum value for the entropy is ln(d), where *d* is the dimension of the Hilbert space). Recall the von Neumann entropy (Equation (22)), where the base of the logarithm is replaced:

$$S_{\mathrm{Von Neumann}}^{\prime }\;=-\mathrm{Tr}\left(\;\rho \log_{2}\left(\rho \right)\;\right)$$

Now, using $\ln\left(x\right)=\log_{2}\left(x\right)/\log_{2}\left(e\right)$ and the linearity of the trace, we obtain

$$S_{\mathrm{Von Neumann}}^{\prime }\;=-\log_{2}\left(e\right)\;\cdot \;\mathrm{Tr}\left(\;\rho \ln(\rho )\;\right)$$

so that the maximal value for the entropy is indeed $\ln\left(2\right)\;\cdot \;\log_{2}\left(e\right)=1$.

#### 5.4.1. Results—Original Dataset

The results for the first dataset are in Table 2. In the non-amplitude-encoded cases, the entropy is (≈28%) lower when forgetting the object than when forgetting the subject for the first sentence type (1). This is contrary to what we expect. As reasoned in Section 5.1, we expect the entropy for the latter case to be lower, because the objects in the original dataset are non-ambiguous, while the subjects are ambiguous. This means that removing the object leaves the sentence more ambiguous, thus with higher entropy. On the other hand, the fidelity of the sentences in which the subject is forgotten is (≈7%) higher than the fidelity of sentences in which we forget the object. This means that the sentences in which we forget the subject are more similar to the target category than those in which we forget the object. We expect this because forgetting the object removes more information about the sentence’s category. Thus, the ambiguity is higher for sentences with a missing subject, yet the sentences are more easily identified with their correct category. The fidelity values themselves are low (0.5 and 0.475 in case of forgetting the subject and object, respectively), indicating low overlap between the correct and the predicted categories. Furthermore, the entropy values are relatively high, especially for the case in which the subject is forgotten (0.825).

For sentence type 2, by adding linguistic information and subsequently disambiguating the sentences, the overall entropy drops significantly (by a factor of around 3 for both cases with the subject and object being forgotten). This behaviour is expected since, for this sentence type, we add words to the sentence to disambiguate it, which makes the sentence overall less ambiguous.

However, the entropy for the case of forgetting the subject remains higher (by a factor of >2) than the entropy for the case of forgetting the object, which, as above, is contrary to our expectations. The fidelity values are very similar between the cases of forgetting the object (0.707) and the subject (0.706) in these sentences. As in the case of the first sentence type (1), we would expect the fidelity of the case in which the subject is forgotten to be slightly higher than is the case for the first sentence type (1). However, as we argue above, we also expect the fidelity to be connected with the entropy values, which is not supported by the results.

Next, we discuss the average entropy and fidelity values for the models in which the noun meanings are amplitude-encoded. We see that for sentence type 1, the entropy in the case of forgetting the subject is lower than in the case of forgetting the object (by a factor of ≈4.6), as expected. Furthermore, we notice that the entropy of the case in which the subject is forgotten is close to one (0.960). An entropy of one would correspond to a maximally mixed state, which indeed makes sense in our case because there is no indication of what category a sentence in which the object is forgotten belongs to since there are only ambiguous subjects in the dataset. This is exactly what the maximally mixed state describes. The fidelity values are slightly higher (≈2% for both object and subject being forgotten) than in the case of the non-amplitude-encoded approach, and the average reported fidelity is slightly higher in cases in which the subject is forgotten than in cases in which the object is forgotten. Again, we would expect the difference to be bigger and the fidelity of the former to be closer to one.

For sentence type 2, we see similar behaviour when considering the approach in which amplitude encoding was used. The overall lowest value for the entropy in the case of forgetting the subject is 0.0198, which is not significantly lower than in the case of no amplitude encoding. This makes sense, as in this structure a verb is present to indicate what category the sentence belongs to. If the non-ambiguous object is present as well, the model is likely to predict the correct category with high confidence. The entropy for the case of the forgotten object is higher than in the non-amplitude-encoded case (by ≈54%). The fidelity values for sentence type 2 remain similar between the cases of forgetting object (0.836) and subject (0.848), where the fidelity for the case of the forgotten subject is slightly higher, which is intuitive, as argued above.

Discussion

Given these results, the following question arises. Why do the approaches in which amplitude encoding is employed report values of average entropy that make significantly more sense than cases in which no amplitude encoding is employed? Firstly, as we saw in Section 4.2.1, the model is not able to make better predictions when the nouns are amplitude-encoded onto one qubit (which they are in this case). This means that the benefit of amplitude encoding is not displayed in the model’s performance measures. However, by amplitude encoding, we not only make the noun space interpretable (by assigning words belonging to different categories to opposite sides of the Bloch sphere), but we maximise the distance between the states representing these words on the Bloch sphere, which makes the encoding itself less ambiguous. Ultimately, a bigger part of the prediction of the category is now dependent on the nouns. This means that the addition or subtraction of linguistic information (adding a word to the sentence or forgetting a word in the sentence) in the form of words encoded to opposite sides of the Bloch sphere has a higher impact on the prediction of the category than if these states to which the words are encoded are similar to each other. Due to this disambiguating effect that amplitude encoding has on the nouns, the subtraction and addition of linguistic information are more reasonably correlated with the entropy in the case of amplitude encoding than in other cases.

This does not explain why the entropy values for the cases in which the subject is forgotten are higher than when the object is forgotten, which is contrary to our expectations. The model trained on the new dataset (Figure 10b) does not clearly encode words that belong to similar categories to similar parts on the Bloch sphere. It might be that the encoding procedure intrinsic to the learning process of this model is something that cannot be intuitively understood. We argue that it is the process of making the noun space interpretable, which connects the entropy to linguistic ambiguity.

#### 5.4.2. Results—New Dataset

All the values, for both the average entropy and average fidelity, as well as the first and second sentence types, are reported in Table 3 for the model trained on the new dataset. For both sentence type 1 and type 2, the entropy is lower than for the original dataset (Table 2): for the first sentence type (1), the entropy values drop from 0.825 and 0.592 to 0.652 and 0.287 for the case of forgetting the subject and the object, respectively. Furthermore, the unintuitive result remains that for sentence type 1, the case of forgetting the object yields lower entropy than the case of forgetting the subject (by a factor of ≈2.3). This problem, as for the original dataset, does not remain when amplitude-encoding the nouns. For sentence type 2, the average entropy of cases in which the object is forgotten (0.0392) is higher than in the case of forgetting the subject (0.0110). This makes sense since the model should be able to categorise sentences based on the verb in the sentence, as argued above. These results are more intuitive than in the approach using the original dataset. The use of amplitude encoding again yields results that are closer to our expectations. Forgetting the subject in sentence type 1 yields a very low entropy (0.0686), while forgetting the object yields an entropy of 0.936. Furthermore, for sentence type 2, the entropies for both the case of forgetting the subject (0.0812) and the case of forgetting the object (0.267) are lowered compared to sentence type 1, as expected.

As for fidelity, the values are very similar to the findings using the original dataset for both sentence types and both applying amplitude encoding and not using amplitude encoding. To conclude, the average values for entropy and fidelity are very similar to the above case in which the original dataset was used. However, there is a notable difference in the performance on the tasks involving sentence type 2. The resulting entropy values, especially using the non-amplitude-encoded approach, are closer to our expectations.

For sentence type 3, we display the average entropy and fidelity in Table 4. Remember that in this approach non-ambiguous subjects are used to disambiguate sentences. For the case of the non-amplitude-encoded approach, the values for the average fidelity (0.730 and 0.486 for forgetting the subject and object, respectively) and entropy (0.443 and 0.217 for forgetting the subject and object, respectively) are similar to type 1 and type 2 in Table 3. We note the reoccurring problem of the average entropy being higher for the cases in which the subject is forgotten than for the cases in which the object is forgotten. The fidelity values are slightly more intuitive than what we have seen in the case of sentence type 2. The fidelity rises when forgetting the subject instead of forgetting the object.

In the case of amplitude encoding and sentence type 3, the average entropy for the case in which the subject is forgotten is almost zero (2.26 × 10^−15^). However, the entropy for forgetting the object is relatively high (0.648). Furthermore, the fidelity for the case of the forgotten subject is 0.597, which, since these values are averages, implies that around half of the sentences that were averaged over were classified correctly, while the others were not. Usually, with a low entropy, one expects a high fidelity, but in this case the model predicts the wrong category in around half of the cases. Furthermore, the entropy is significantly higher when using amplitude encoding for the case of forgetting the object compared to the non-amplitude-encoding approach (0.217). From the data presented in this section, we cannot conclude that the process of amplitude encoding decreases the performance when investigating disambiguating effects, as in sentence type 3. We report results for both non-amplitude-encoded and amplitude-encoded cases that are contrary to our expectations. Again, we emphasise that the performance of the models is not directly correlated with interpretability in terms of the connection between ambiguity and entropy. While word representations may effectively capture word meaning in relation to the words and sentences within the training data, examining interpretability to uncover implicit connections in the data is not necessarily correlated with the performance during training.

Discussion

Analysing type 1 and type 2 yields very similar results for the model trained on the new dataset as for the model trained on the original dataset. Overall, the output values of the amplitude-encoded model trained on the new dataset are the most intuitive for type 1 and type 2. This might be due to the advantage of amplitude encoding being maximised when training using the new dataset, because there is more variation in the data due to the non-ambiguous subject programmer and chef. Although the explicit words chef and programmer are not used in type 1 and type 2 sentence types (only ambiguous subjects are used), the representations learned for other words might be more suited to the task due to the verbs and adjectives being forced to generalise better.

Therefore, by amplitude encoding, we endow the model with the ability to disambiguate using nouns. Our results suggest that the model is not able to display these effects when amplitude encoding is not employed. This means that by amplitude encoding, we provide the model with a relation between the meaning of a noun and the actual category. As seen in Section 4.2.1, amplitude encoding does not affect the model’s ultimate performance regarding the convergence or metrics. However, the results suggest that amplitude encoding indeed affects the model’s reasoning abilities. We argue that the convergence of the model, together with the resulting performance metrics, is not the most suitable way to argue about the quality of the model’s training result when reasoning with the learned model.

**Figure 22:**
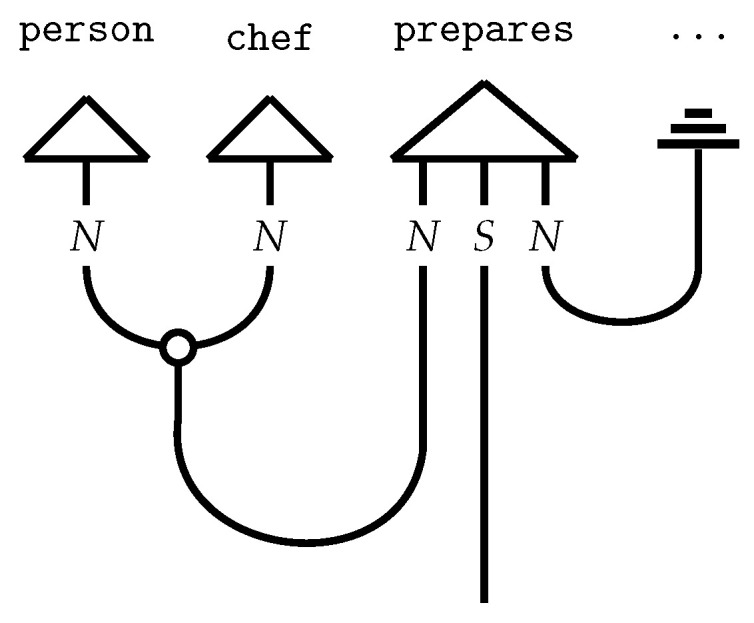
The diagram encoding the meaning of the sentence person prepares … and chef does too., which can be read as person and chef both prepare ….

Analysing sentence type 3 yields results that are contrary to our expectations. Although we do not know what causes the counter-intuitive results when disambiguating sentences (as discussed in Section 5.4.2), this might be related to the construction of the quantum circuit for sentence type 3. As seen in Figure 22, the two nouns in the sentence are entangled before being entangled with the verb. Here, the model learns the verb, which now is provided with an already entangled qubit as the subject. Providing the disambiguating word (e.g., chef) as an entangled qubit, with the qubit representing the ambiguous noun chef, might introduce effects that are beyond our intuition. Overall, the model’s performance on this task using sentence type 3 is not as we expected.

## 6. Conclusions and Further Work

We want to stress again that QNLP should not be expected to outperform NLP in general just yet. However, as Coecke and Kissinger [36] argue, language is inherently quantum. In our work, we show that combining classical data and quantum machine learning algorithms yields promising results. However, the huge amounts of data used for large language models alone are far beyond the recent state of quantum algorithms. First, we reproduced and extended a task by Lorenz et al. [3] and reproduced their results. We continued by investigating the application of encoding classical data onto a quantum computer using amplitude encoding. Our results suggest that using amplitude encoding increases the model’s performance when encoding the noun meanings onto two qubits. However, amplitude encoding does not substantially affect the model’s performance when encoding noun meanings onto one qubit.

In the last contribution, we addressed the relation between mixedness in density matrices and linguistic ambiguity in the sentences they represent. Overall, the results suggest that there is a correlation between linguistic ambiguity and entropy in a quantum circuit. We see that the relation between the two quantities is more intuitive when amplitude encoding is employed in the training process of the model. This can be explained by the fact that amplitude-encoding noun meanings makes the noun space classically interpretable. Future research may explore the general impact of introducing classical data via amplitude encoding on the performance of quantum machine learning models. This would require averaging the performances of several models, as addressed in Section 3.2. Further, the performance metrics and loss curves are not directly linked to interpretability in terms of the relation between the entropy of a density matrix and the ambiguity in the sentence it represents. Future research will focus on exploring the relationship between performance metrics and interpretability. This can be achieved by executing the model multiple times with different initial parameter settings and assessing the interpretability of the results for each individual run. Based on the contributions of this work, it is worth investigating the connection between variations in entropy and ambiguity more profoundly. One might choose multi-qubit encodings. The performance of models where the noun meaning is encoded onto three qubits (making the noun space eight-dimensional) can be investigated. As argued, Figure 15 shows that by moving from four to eight dimensions, the amount of lost information is substantially reduced.

## Figures and Tables

**Table 1:** Metrics for the pennylane and NumPy models trained using different datasets as well as one- and two-qubit approaches, where $N_{q}$ denotes the number of qubits on which a noun is encoded.

Model	$N_{q}$	Dataset	Accuracy	κ	$F_{1}$-Score
pennylane	1	original	0.97	1.00	1.00
pennylane	1	new	0.97	1.00	1.00
NumPy	1	original	0.90	0.87	0.94
NumPy	1	new	0.90	0.87	0.94
pennylane	2	original	1.00	1.00	1.00
pennylane	2	new	1.00	1.00	1.00
NumPy	2	original	0.82	0.63	0.81
NumPy	2	new	0.80	0.60	0.79

**Table 2:** Average entropy and fidelity measures (determined as explained in the main text) for the original dataset, and the first and second sentence types (1) and (2).

Original Dataset	Average Entropy	Average Fidelity
Sentence type (1):		
subject prepares object		
Forget subject	0.825	0.507
Forget object	0.592	0.475
Forget subject, amplitude-encoded	0.210	0.516
Forget object, amplitude-encoded	0.960	0.493
Sentence type (2):		
subject prepares object and verb* it		
Forget subject	0.302	0.706
Forget object	0.144	0.707
Forget subject, amplitude-encoded	0.0198	0.848
Forget object, amplitude-encoded	0.222	0.836

**Table 3:** Average entropy and fidelity values (determined as explained in the main text) for the new dataset for sentence types 1 and 2 for cases of forgetting object or subject in the sentences under investigation, with amplitude encoding employed when indicated.

New Dataset	Average Entropy	Average Fidelity
Sentence type 1:		
subject prepares object		
Forget subject	0.652	0.488
Forget object	0.287	0.428
Forget subject, amplitude-encoded	0.0686	0.594
Forget object, amplitude-encoded	0.936	0.492
Sentence type 2:		
subject prepares object and verb* it		
Forget subject	0.0110	0.990
Forget object	0.0392	0.971
Forget subject, amplitude-encoded	0.0812	0.628
Forget object, amplitude-encoded	0.267	0.694

**Table 4:** Average entropy and fidelity measures (determined as explained in the main text) for the new dataset for the third sentence type (3) and cases of forgetting object or subject in the sentences under investigation, with amplitude encoding employed when indicated.

New Dataset	Average Entropy	Average Fidelity
Sentence type 3:		
subject prepares object, subject* does too		
Forget subject	0.443	0.730
Forget object	0.217	0.486
Forget subject, amplitude-encoded	2.26 × 10^−15^	0.579
Forget object, amplitude-encoded	0.648	0.492

**Figure A1:**
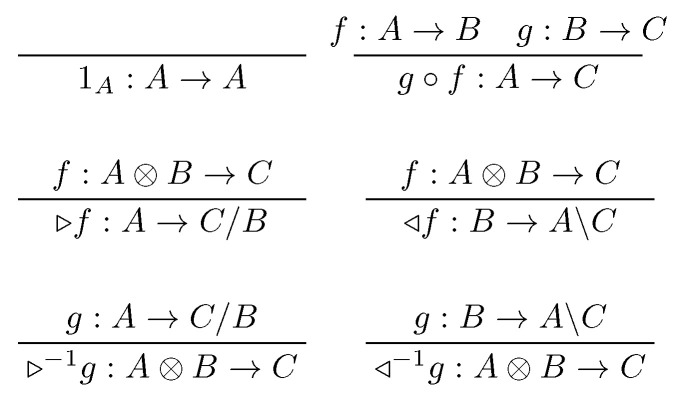
The Lambek calculus as a deductive system [65].

**Figure A2:**
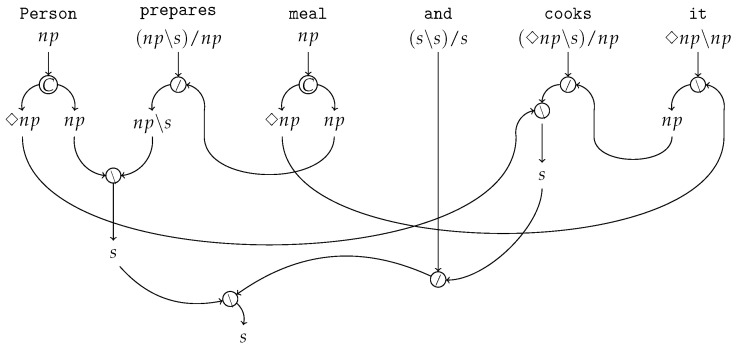
The proof net encoding the meaning of the sentence person prepares meal and cooks it.

**Figure A4:**
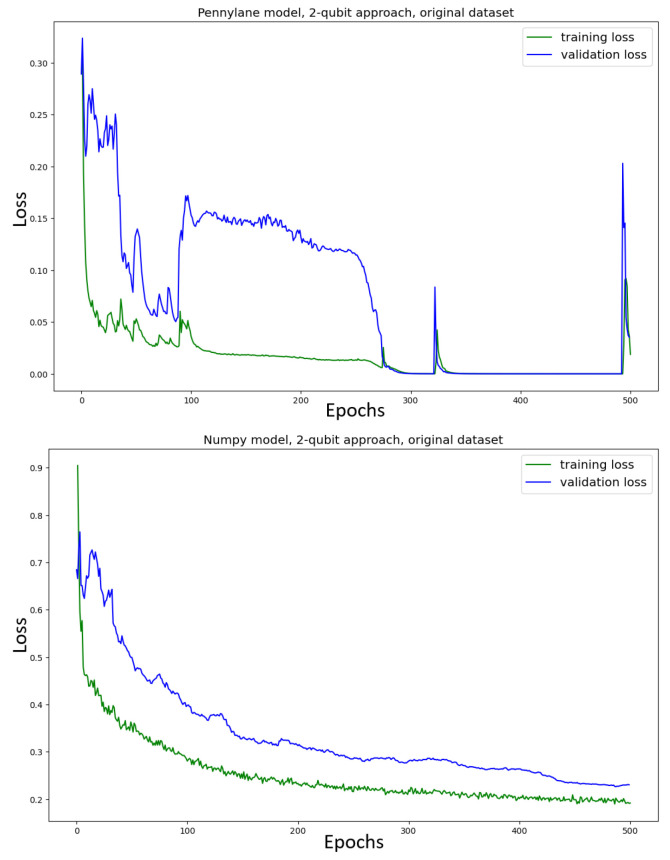
Loss curves for training and validation data for the pennylane and NumPy models, with noun meanings encoded on two qubits using the original dataset. Metrics, left: accuracy = 1.00, κ = 1.00, F_1_-score = 1.00. Metrics, right: accuracy = 0.820, κ = 0.630, F_1_-score = 0.810.

## Data Availability

Data is contained within the article.

## Appendix A. Lambek Calculus and Proof Nets

In Lambek calculus, as well as in the pregroup formalism, words are assigned types. In contrast to the pregroups discussed above, the Lambek calculus is a type-logical grammar. The sequence of types assigned to words induces a proof if the sequence is grammatical. The (non-associative) Lambek calculus can be presented as a deductive system, where the connectives ⊗,∖, and / are introduced (Figure A1).

The Lambek calculus can be shown to be a valid grammar formalism to replace the pregroup grammar in the DisCoCat framework, as Coecke et al. [24] argue.

The relation between the pregroup grammar and the Lambek calculus is that derivations in the former can be seen as the *image* of the derivations in the latter, as argued by Wijnholds [14], based on original work by Buszkowski [66], who gives a translation from the types in Lambek calculus to pregroup types as

(A1)

Wijnholds [67] introduces a graphical way of displaying composition in the Lambek calculus via *proof nets*, similar to string diagrams in the pregroup grammar. A *constructor link* and a *destructor link* are introduced for each connective in the calculus. Destructor links are defined as

while constructor links are

The running example of Alice plays guitar is

Furthermore, to model verb phrase ellipsis, Wijnholds [14] uses a multimodal extension to the Lambek calculus with control modalities, called ${\mathrm{NL}}_{⋄}$, originally introduced by Moot [68]. The Lambek calculus is extended by two unary connectives ⋄ and □, which obey

(A2) ⋄A→BifandonlyifA→□B

where *A* and *B* are types. This structure, together with a number of morphisms (among them *C*, seen in Figure A2), allows for the *controlled copying* of words. For an extensive overview of the systems, we refer to Wijnholds [14].

The sentence Person prepares meal and cooks it can be modelled in terms of proof nets (Figure A2), where the words person and meal are copied using the morphism *C* and the copies are moved to the appropriate place in the sentence (to the verb cooks, guided by the assignment of the control modality ⋄ to the verb cooks: (⋄np∖s)/np). The crossing wires in the above diagram reflect the (controlled) commutative nature of the Lambek calculus with control modalities. This yields the DisCoCat diagram in Figure 20. The same can be derived using, e.g., the Lambek calculus with soft sub-exponentials SSLM [18] (the copying map would be a 2-projection from the corresponding truncated Fock space [19] N⊕(N⊗N)⊕…⊕(N⊗N⊗…⊗N)).

## Appendix B. Plots

The plots referenced in the main text are collected here both for Section 3 and Section 4.

## Appendix C. Dataset

**Table A1:** The original dataset presented in Section 3.1. In the new dataset, the words man and woman are replaced with the words chef and programmer.

woman prepares tasty dinner	woman cooks tasty sauce
skillful man prepares dinner	skillful woman cooks sauce
man prepares sauce	person cooks tasty sauce
man cooks sauce	woman bakes meal
skillful man bakes sauce	person bakes meal
skillful woman bakes dinner	skillful woman cooks dinner
man cooks meal	woman bakes sauce
woman prepares meal	skillful man prepares sauce
skillful man bakes dinner	woman cooks tasty meal
man prepares meal	woman prepares tasty meal
woman prepares sauce	woman prepares dinner
skillful person prepares meal	skillful person bakes dinner
skillful woman bakes meal	man bakes tasty meal
person prepares tasty meal	man bakes tasty dinner
skillful man cooks dinner	person cooks dinner
skillful woman prepares meal	skillful woman bakes sauce
skillful man bakes meal	woman cooks meal
woman bakes dinner	skillful man cooks meal
man cooks dinner	woman cooks tasty dinner
woman cooks dinner	man bakes tasty sauce
man prepares dinner	skillful person cooks sauce
person prepares tasty sauce	skillful person bakes sauce
skillful man cooks sauce	woman bakes tasty meal
person cooks meal	person bakes tasty sauce
person bakes dinner	man cooks tasty meal
skillful person cooks meal	person cooks sauce
man cooks tasty sauce	skillful person bakes meal
man prepares tasty meal	man prepares tasty sauce
person bakes tasty meal	person prepares dinner
man bakes sauce	person cooks tasty dinner
woman bakes tasty sauce	skillful person prepares sauce
person prepares tasty dinner	woman bakes tasty dinner
woman cooks sauce	skillful woman prepares software
woman runs useful program	skillful person runs software
skillful person prepares program	man prepares program
skillful person prepares software	man prepares useful software
woman debugs program	skillful woman runs application
man debugs software	skillful woman debugs application
person debugs software	woman runs useful software
person debugs program	skillful woman debugs software
skillful woman debugs program	person runs program
person runs useful application	woman runs useful application
woman runs application	man prepares software
person prepares useful program	man debugs useful application
person debugs useful application	woman prepares program
man prepares useful application	man debugs useful software
man prepares application	person debugs useful software
person runs application	woman runs program
skillful man prepares program	woman runs software
skillful man debugs software	skillful man prepares software
person prepares software	person runs software
man debugs program	man runs software
person prepares useful application	woman debugs software
skillful man runs software	woman debugs application
woman debugs useful program	skillful woman runs program
person runs useful program	skillful person prepares application
man prepares useful program	man runs program
woman prepares software	person prepares useful software
skillful person debugs program	person debugs application
skillful person debugs software	skillful woman runs software
person debugs useful program	man runs application
woman debugs useful software	man runs useful application
person prepares program	woman debugs useful application
skillful woman prepares application	man debugs application
woman prepares useful application	man debugs useful program
